# Effect of the immune cells and plasma metabolites on rheumatoid arthritis: a mediated mendelian randomization study

**DOI:** 10.3389/fendo.2024.1438097

**Published:** 2024-09-03

**Authors:** Qi-Pei Liu, Hong-Cheng Du, Ping-Jin Xie, Sheng-Ting Chai

**Affiliations:** ^1^ The Third School of Clinical Medicine, Guangzhou University of Chinese Medicine, Guangzhou, China; ^2^ Graduate School of Guangzhou University of Chinese Medicine, Guangzhou, China; ^3^ Graduate School of Guangxi University of Chinese Medicine, Nanning, China; ^4^ Shenzhen Hospital of Shanghai University of Traditional Chinese Medicine, Shenzhen, China; ^5^ Department of Arthrosis, the Third Affiliated Hospital of Guangzhou University of Chinese Medicine, Guangzhou, China

**Keywords:** immune cells, plasma metabolites, rheumatoid arthritis, bidirectional causality, mediation analysis, causality

## Abstract

**Background:**

Increasing evidence indicates a close relationship between alterations in human immune cells and plasma metabolites with Rheumatoid Arthritis (RA). However, limited studies have left the causal relationships behind these links unclear.

**Methods:**

A bidirectional Mendelian Randomization (MR) study was conducted, combined with mediation analysis, using data from genome-wide association study database covering 731 immune cell phenotypes and 1,400 plasma metabolite traits to explore their causal relationships with RA and potential mediating effects. The primary method used for MR analysis was inverse-variance weighted and False Discovery Rate (FDR) correction was applied to verify the robustness of our results.

**Results:**

HLA DR on CD33- HLA DR+ (myeloid cell group) (OR, 1.422; 95% CI, 1.194–1.694; P < 0.001; P_FDR_ = 0.012) increased the risk of developing RA. CD19 on IgD+ CD38- naive (B cell group) (OR, 0.969; 95% CI, 0.954–0.985; P < 0.001; P_FDR_ = 0.021) reduced the risk of developing RA. RA was a risk factor for HLA DR on CD14- CD16+ monocytes (monocyte group) (OR, 1.242; 95% CI, 1.102–1.401; P < 0.001; P_FDR_  = 0.047). RA was a protective factor for memory B cell %lymphocyte (B cell group) (OR, 0.861; 95% CI, 0.795–0.933; P < 0.001; P_FDR_ = 0.050), CD4+ CD8dim T cell %lymphocyte (TBNK group) (OR, 0.802; 95% CI, 0.711–0.904; P < 0.001; P_FDR_  = 0.043), CD4+ CD8dim T cell %leukocyte (TBNK group) (OR, 0.814; 95% CI, 0.726–0.913; P < 0.001; P_FDR_  = 0.046), CD24 on IgD+ CD24+ B cells (B cell group) (OR, 0.857; 95% CI, 0.793–0.927; P < 0.001; P_FDR_  = 0.038), and CD24 on unswitched memory B cells (B cell group) (OR, 0.867; 95% CI, 0.797–0.942; P < 0.001; P_FDR_  = 0.050). Increasing levels of docosatrienoate (22:3n3) (OR, 0.886; 95% CI, 0.838–0.936; P < 0.001; P_FDR_ = 0.023) significantly reduced the risk of developing RA. The mediating effect of plasma metabolites in this context was not established.

**Conclusion:**

This study provides genetic evidence for the intricate relationships between immune cells, plasma metabolites, and RA, highlighting the potential mechanisms involved. This will contribute to future directions in precision medicine and research.

## Introduction

Rheumatoid Arthritis (RA) is a chronic autoimmune disease characterized by the disruptions of multiple metabolic pathways (1). It features immune cell infiltration, systemic inflammatory responses, neovascularization, and synovial hyperplasia, leading to bone and cartilage erosion and degradation, which subsequently results in loss of function and disability in patients (1, 2). Annually, 0.46% of the global population is affected by RA, with women being approximately three times more likely to develop the condition than men (3). About 2.3 million Europeans are diagnosed with RA annually, with a prevalence of approximately 1% (4, 5). The direct and indirect socioeconomic costs of RA in European countries exceed €45 billion annually (4). RA has a significant negative impact on patients’ economic burden, mental health, and quality of life, making it crucial to enhance research for early diagnosis and treatment.

The immune mechanisms in patients with RA are complex, involving not only immune diseases but also potentially affecting the respiratory system, bone and soft tissues, psychology, and cardiovascular areas (6, 7). For instance, immune cells, such as B cells and T cells, in patients with RA produce pro-inflammatory factors that stimulate fibroblast polarization, activating osteoclasts and releasing matrix metalloproteinases, ultimately driving bone and cartilage destruction (8). Recent studies have indicated that the aging of immune cells leads to protein homeostasis failure, mitochondrial dysfunction, and lysosomal failure, resulting in impaired cellular functions that trigger autoimmunity and ultimately induce RA (9).

Metabolomics enables high-throughput characterization of cells, body fluids, and tissues within an organism, thereby revealing physiological and pathological changes and enhancing the understanding of diseases (10, 11). In systemic diseases such as rheumatoid arthritis, abnormal changes in circulating metabolomics may reflect the patient’s microbiome, complications, genetic susceptibility, drug responses, systemic inflammation, and various environmental factors such as smoking, alcohol consumption, and diet (12). Metabolites serve as reference indicators for the effects of internal and external factors on a biological organism, such as elevated plasma cholesterol levels in patients with hypercholesterolemia. Metabolic changes in patients with RA are complex and diverse. For example, the upregulation of branched-chain amino acids and alanine and downregulation of glucose and glycylglycine play direct or indirect roles in inducing immune responses and driving inflammatory mechanisms in patients (13). Enhancing metabolomic research in patients with RA can help us better understand drug mechanisms and aid clinicians in improving diagnosis and treatment prognosis by recognizing variations in internal metabolites among patients.

Mendelian Randomization (MR) is an epidemiological method based on Mendel’s laws, which uses genetic variations as tools to explore causal relationships between risk factors and outcomes (14). Compared with other statistical methods, MR can minimize the influence of potential confounders and reverse causation, making the results more reliable (15). Therefore, to explore the potential causal relationships between immune cell phenotypes, plasma metabolites, and RA, two-sample bidirectional MR analysis and mediation analysis were performed using Genome-Wide Association Study (GWAS) data and FinnGen database.

## Materials and methods

### Study design

First, the possible bidirectional causal relationships between immune cells and RA were explored. Next, the potential bidirectional causal relationships between metabolites and RA were investigated. Plasma metabolites were then introduced as mediators to examine their mediating effects on the relationship between immune cell phenotypes and RA. All included Instrumental Variables (IVs) met three basic assumptions: first, IVs are significantly associated with exposure; second, IVs are not related to confounders or outcome; third, the included IVs influence the outcome solely through their effect on exposure (16).

### Data source

#### GWAS data sources for immune cells

The GWAS data for the 731 immune cell phenotypes used in this study were obtained from a public repository (GCST0001391 to GCST0002121) (17, 18). The dataset included 22 million genetic variants from 3,757 individuals of Italian Sardinian ancestry. The dataset was categorized into 118 absolute cell counts, 389 median fluorescence intensities representing surface antigen levels, 32 morphological phenotypes, and 192 relative cell counts.

#### GWAS data sources for plasma metabolites

The GWAS data for the 1400 plasma metabolites used in this study were obtained from a public repository (GCST90199621 to GCST90201020) (18, 19). The dataset included more than 8,000 individuals of European ancestry and plasma metabolite data consisting of 1,091 blood metabolites and 309 metabolite ratios.

#### Data source for RA

Data related to RA was sourced from the tenth edition of the FinnGen database (R10) (20, 21). The dataset finngen_R10_M13_RHEUMA included 276,565 study subjects of European descent, comprising 13,621 cases and 262,844 controls.

### Selection of instrumental variables

First, for immune cells and plasma metabolites, the significance threshold was set to P < 1*10^^-5^, in line with previous studies (22). For reverse MR, a more stringent threshold (P < 5*10^^-8^) was applied to select RA-related Single Nucleotide Polymorphisms (SNPs) as instrumental variables. Second, strongly linked variants were excluded to avoid linkage disequilibrium issues among SNPs (R2 = 0.001, clumping distance = 10,000 kb). Third, to avoid weak instrument bias, F-statistics were calculated for all IVs included in the study and the IVs with F < 10 were excluded (23).

### Statistical analysis

The Inverse-Variance Weighted (IVW) method with a random-effects model was used as the primary analysis method for the MR analysis. To ensure the reliability of the results, the P-values obtained from the IVW method were corrected using the Benjamini–Hochberg method to control the False Discovery Rate (FDR) due to multiple tests (24). Considering that the accuracy of the IVW method is based on the assumption of no horizontal pleiotropy, MR-Egger regression was performed throughout the MR process to calculate the intercept and assess the significance of horizontal pleiotropy (25). In the absence of horizontal pleiotropy, a P_FDR_ <0.05 indicates significant positive results, and a P_FDR_ <0.2 represents suggestive positive results (26). A two-step MR design was used to perform mediation analysis to determine whether plasma metabolites mediate the relationship between immune cells and RA. Heterogeneity between IVs was assessed by Cochran’s Q test based on IVW and MR-Egger methods. Funnel plots, Leave-One-Out (LOO) analysis, and additional methods such as MR-Egger, weighted median, weighted mode, and simple mode were included as part of our sensitivity analysis throughout the study to enhance the robustness of the results.

All data analyses were performed using the “TwoSampleMR” package (version 0.5.10) in R statistical software (version 4.3.3).

## Results

### Results of selection of instrumental variables

For detailed information on IVs and their association with immune cell phenotypes, blood metabolites, and rheumatoid arthritis, please refer to **Supplementary Data S1**.

### Effects of immune cells on RA risk

**Figure 1:**
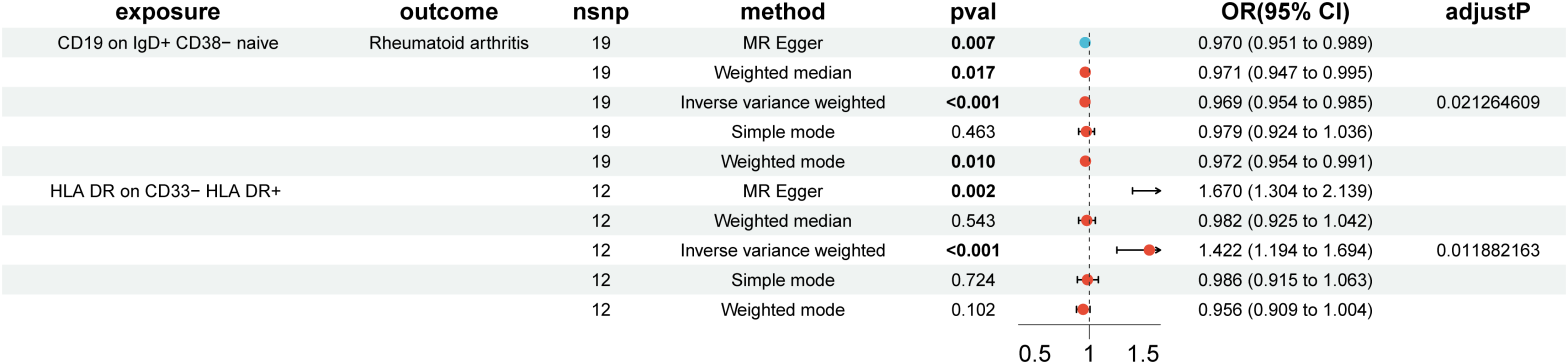
Forest plot of positive causal associations between immune cell phenotypes and rheumatoid arthritis after false discovery rate correction.

**Figure 2:**
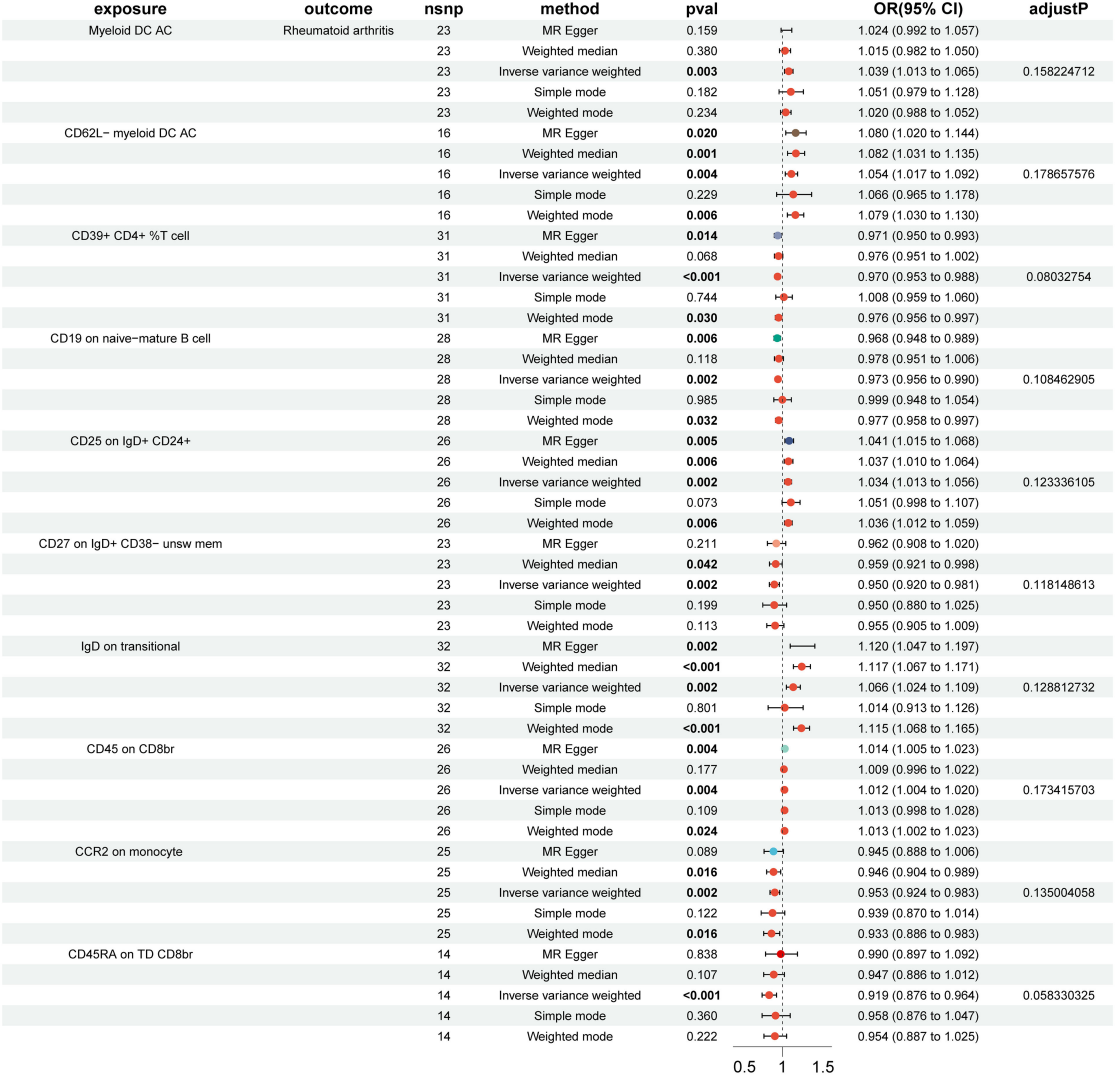
Forest plot of suggestive positive causal associations between immune cell phenotypes and rheumatoid arthritis after false discovery rate correction.

In the MR analysis of immune cells and RA risk, this study found that 57 immune cell phenotypes were significant in IVW method (P < 0.05) and showed no horizontal pleiotropy in MR-Egger regression (**Supplementary Figure S1**). After FDR adjustment, two immune phenotypes were identified with a significant causal relationship with RA (**Figure 1**, **Supplementary Figure S2**). HLA DR on CD33- HLA DR+ (myeloid cell group) (Odds Ratio [OR], 1.422; 95% Confidence Interval [CI], 1.194–1.694; P < 0.001; P_FDR_ = 0.012) increased RA risk (**Supplementary Figure S3**). CD19 on IgD+ CD38- naive (B cell group) (OR, 0.969; 95% CI, 0.954–0.985; P < 0.001; P_FDR_ = 0.021) decreased RA risk (**Supplementary Figure S3**). The robustness of the outcome was further validated through LOO analysis and funnel plots (**Supplementary Figures S4**, **S5**). After FDR adjustment, 10 immune cell phenotypes showed suggestive causal relationships with RA (**Figure 2**). The results suggested that Myeloid DC AC (OR, 1.389; 95% CI, 1.013–1.065; P = 0.003; P_FDR_ = 0.158), CD62L- myeloid DC AC (OR, 1.054; 95% CI, 1.017–1.092; P = 0.002; P_FDR_ = 0.179), CD25 on IgD+ CD24+ (OR, 1.034; 95% CI, 1.013–1.056; P = 0.004; P_FDR_ = 0.123), IgD on transitional (OR, 1.066; 95% CI, 1.024–1.109; P = 0.002; P_FDR_ = 0.129), and CD45 on CD8br (OR, 1.012; 95% CI, 1.004–1.020; P = 0.004; P_FDR_ = 0.173) may increase RA risk (**Figure 2**). CD39+ CD4+ %T cell (OR, 0.970; 95% CI, 0.953–0.988; P < 0.001; P_FDR_ = 0.080), CD19 on naive-mature B cell (OR, 0.973; 95% CI, 0.956–0.990; P = 0.002; P_FDR_ = 0.108), CD27 on IgD+ CD38- unswitched memory (OR, 0.950; 95% CI, 0.920–0.981; P = 0.002; P_FDR_ = 0.118), CCR2 on monocyte (OR, 0.953; 95% CI, 0.924–0.983; P = 0.002; P_FDR_ = 0.135), and CD45RA on TD CD8br (OR, 0.919; 95% CI, 0.876–0.964; P < 0.001; P_FDR_ = 0.058) may decrease RA risk (**Figure 2**). The heterogeneity of IVs in the MR analysis and horizontal pleiotropy in MR-Egger regression for immune phenotypes and RA are shown in **Supplementary Data S2**.

### Impact of RA on immune cells causation

**Figure 3:**
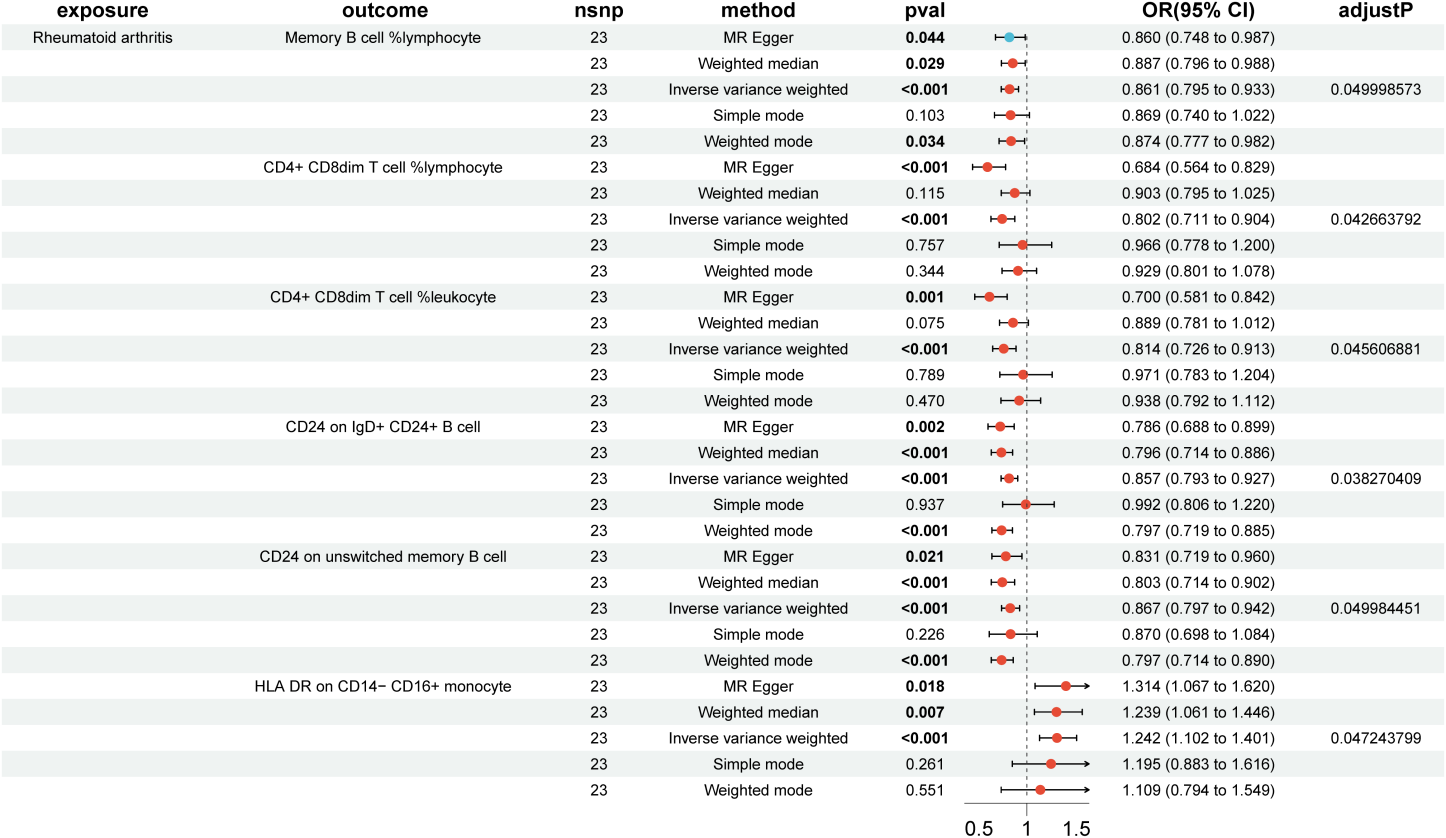
Forest plot of positive causal associations between rheumatoid arthritis and immune cell phenotypes after false discovery rate correction.

**Figure 4:**
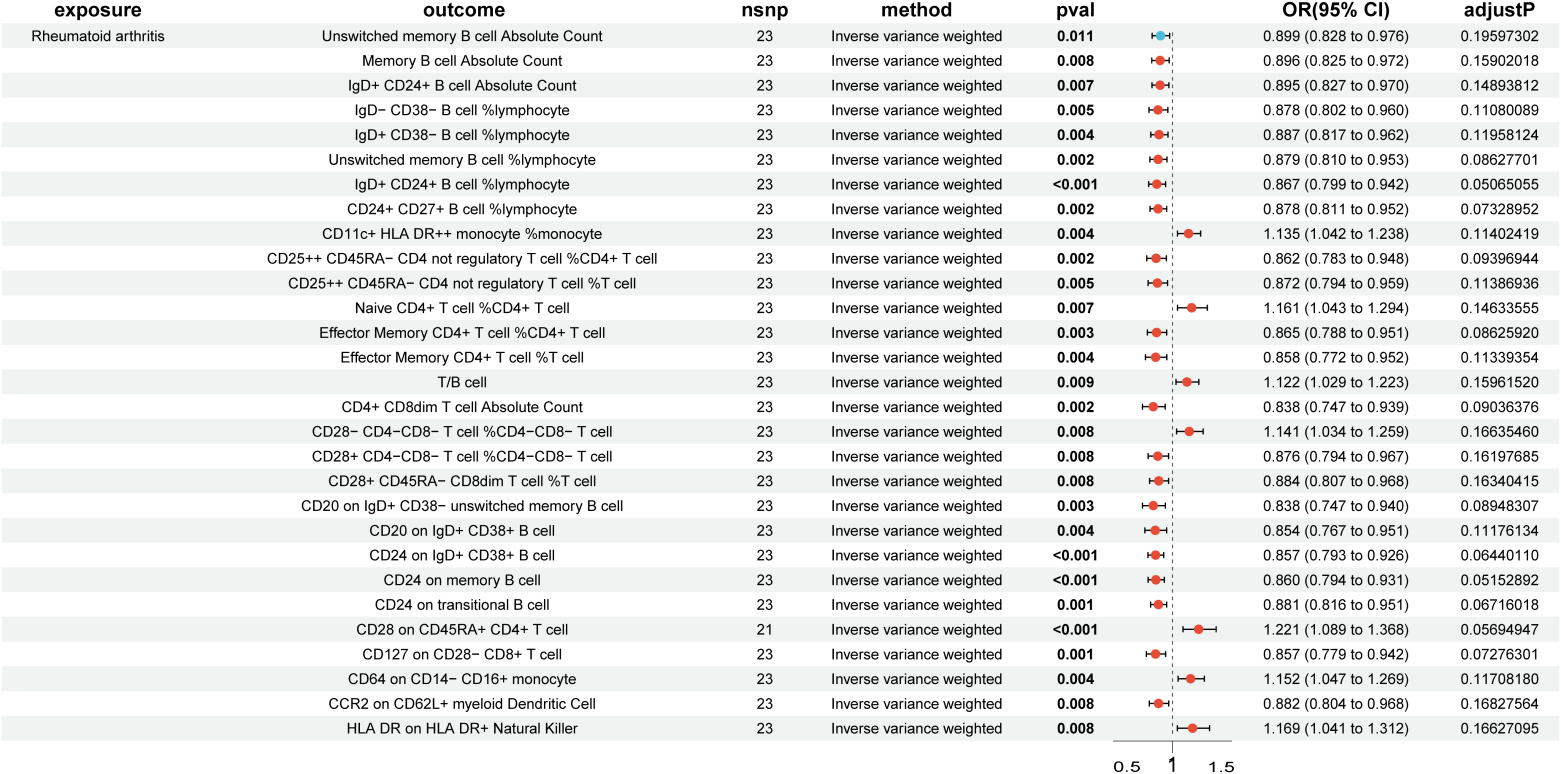
Forest plot of suggestive positive causal associations between rheumatoid arthritis and immune cell phenotypes after false discovery rate correction.

In the reverse MR analysis, 84 immune cell phenotypes were significant in IVW method (P < 0.05) and showed no horizontal pleiotropy in MR-Egger regression (**Supplementary Figure S6**). After FDR adjustment, the results showed that RA had significant causal relationships with six immune cell phenotypes (**Figure 3**, **Supplementary Figure S7**). RA was found to be a risk factor for HLA DR on CD14- CD16+ monocytes (monocyte group) (OR, 1.242; 95% CI, 1.102–1.401; P < 0.001; P_FDR_ = 0.047) (**Supplementary Figure S8**). RA was found to be a protective factor for Memory B cell %lymphocyte (B cell group) (OR, 0.861; 95% CI, 0.795–0.933; P < 0.001; P_FDR_ = 0.050), CD4+ CD8dim T cell %lymphocyte (TBNK group) (OR, 0.802; 95% CI, 0.711–0.904; P < 0.001; P_FDR_ = 0.043), CD4+ CD8dim T cell %leukocyte (TBNK group) (OR, 0.814; 95% CI, 0.726–0.913; P < 0.001; P_FDR_ = 0.046), CD24 on IgD+ CD24+ B cell (B cell group) (OR, 0.857; 95% CI, 0.793–0.927; P < 0.001; P_FDR_ = 0.038), and CD24 on unswitched memory B cell (B cell group) (OR, 0.867; 95% CI, 0.797–0.942; P < 0.001; P_FDR_ = 0.050) (**Supplementary Figure S8**). The outcome was further validated through LOO analysis and funnel plots (**Supplementary Figure S9**, **S10**). After FDR adjustment, RA showed suggestive causal relationships with 29 immune cell phenotypes (**Figure 4**). The heterogeneity of IVs in the MR analysis and horizontal pleiotropy in MR-Egger regression for RA and immune phenotypes are shown in **Supplementary Data S2**.

### Effects of plasma metabolites on RA risk

In the MR analysis of 1400 metabolites and RA risk, 59 metabolites were significant in IVW method (P < 0.05) and showed no horizontal pleiotropy in MR-Egger regression (**Supplementary Figure S11**). After FDR correction, an increase in docosatrienoate (22:3n3) levels (OR, 0.886; 95% CI, 0.838–0.936; P < 0.001; P_FDR_ = 0.023) significantly reduced the risk of developing RA. The four other **Supplementary Methods** showed the same trend (**Supplementary Figure S12**). The robustness of these results was further validated by LOO analysis and funnel plots (**Supplementary Figure S12**). The heterogeneity and horizontal pleiotropy results are shown in **Supplementary Data S2**.

### Impact of RA on plasma metabolites causation

In the MR analysis of RA and docosatrienoate (22:3n3) levels, no significant causal relationship was found (OR, 0.997; 95% CI, 0.949–1.048; P = 0.907) (**Supplementary Figure S13**). The heterogeneity and horizontal pleiotropy results are shown in **Supplementary Data S2**.

### Mediation analysis

**Figure 5:**
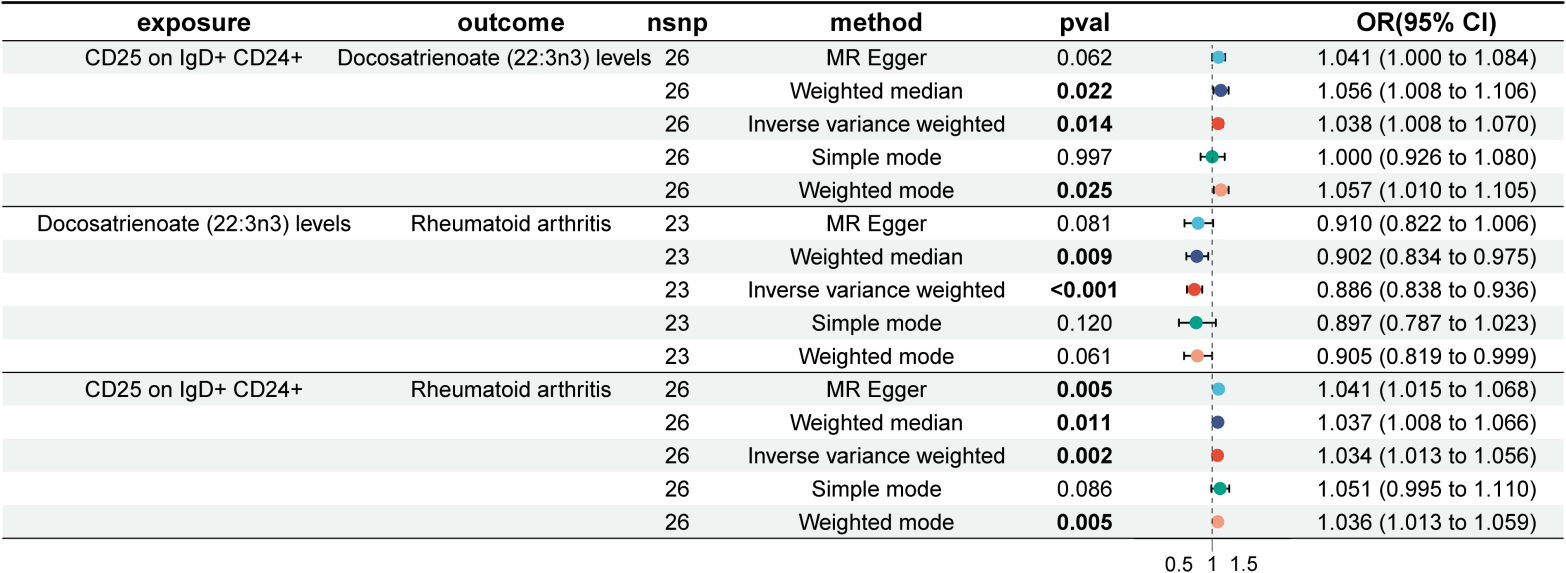
Forest plot of mediation effect analysis of immune cells, plasma metabolites, and rheumatoid arthritis.

First, MR analysis was conducted on the two immune cells, HLA DR on CD33- HLA DR+ and CD19 on IgD+ CD38- naïve, which were FDR-positive in the MR analysis of immune cells and RA, in relation to the metabolite docosatrienoate (22:3n3) levels, but no significant causal relationship was found. Therefore, MR analysis was performed on the suggested positive immune cells: Myeloid DC AC, CD62L- myeloid DC AC, CD25 on IgD+ CD24+, IgD on transitional, CD45 on CD8br, CD39+ CD4+ %T cell, CD19 on naive-mature B cell, CD27 on IgD+ CD38- unswitched memory, CCR2 on monocyte, and CD45RA on TD CD8br in relation to docosatrienoate (22:3n3) levels. It was found that CD25 on IgD+ CD24+ significantly increased docosatrienoate (22:3n3) levels (OR, 1.038; 95% CI, 1.008–1.070; P = 0.014) (**Supplementary Figure S14**). Docosatrienoate (22:3n3) significantly reduces the risk of RA (**Supplementary Figure S15**). However, CD25 on IgD+ CD24+ significantly increases the risk of RA (**Supplementary Figure S16**). The mediation effect of docosatrienoate (22:3n3) levels was not established (**Figure 5**). The heterogeneity and horizontal pleiotropy results are shown in **Supplementary Data S2**.

## Discussion

This is the first study to utilize a large volume of publicly available genetic data to investigate the causal associations between 731 immune cell phenotypes, 1400 plasma metabolites, and RA using MR analysis. Ultimately, it was observed that RA was causally associated with 6 immune cell phenotypes, whereas 2 immune cells phenotypes were causally associated with RA (P_FDR_ < 0.05). Meanwhile, RA was suggestively causally associated with 29 immune cell phenotypes, whereas 10 immune cell phenotypes were suggestively causally associated with RA (P_FDR_ < 0.2). One metabolite was significantly causally associated with the risk of RA (P_FDR_ < 0.05). However, no mediation effect of the metabolite between immune cell traits and RA was observed.

A significant decrease in the risk of RA was observed with a reduction in the proportion of CD19 on IgD+ CD38- naïve cells. B cells are involved in RA through the production of autoantibodies antigen presentation to T cells, and secretion of cytokines (such as tumor necrosis factor alpha, Interleukin-1β) (6, 27). Chimeric antigen receptor T cells have been widely used to treat autoimmune diseases by inducing rapid and sustained depletion of circulating B cells (28). Despite the improvement in inflammatory markers in patients with RA following B cell depletion therapy, the risk of infection and cancer has increased (29). Therefore, it is crucial to distinguish between protective and pathogenic B cells for targeted intervention. Currently, there is no literature on the association between CD19 on IgD+ CD38- naïve cells and the risk of RA. This study is the first to identify a negative causal relationship between CD19 on IgD+ CD38- naïve cells and RA risk, potentially providing new insights into the mechanism of B cell involvement in RA. Our results indicated that an increased proportion of HLA DR on CD33- HLA DR+ cells is associated with an increased risk of RA. The Human Leukocyte Antigen-DR (HLA-DR) molecule presents exogenous antigens to CD4+ T cells, promoting CD40L expression and specific Interferon-γ (IFN-γ) activation on CD4+ T cells, thereby inducing immune responses and contributing to RA development (30, 31). Recent studies have confirmed the Shared Epitope (SE) hypothesis, suggesting that HLA-DR molecules with SE motifs increase the risk of RA by specifically binding to citrullinated peptides and triggering autoimmune processes (32). The HLA-DRB1 *0404 gene is considered to have the greatest impact on increasing the risk of RA (31). Additionally, the presence of SE alleles specifically increases the risk of anti-citrullinated protein antibodies in patients with RA, but the underlying mechanism remains unclear and warrants further investigation (31).

This study revealed a positive correlation between RA and the levels of HLA DR on CD14- CD16+ monocytes. Monocytes, as part of the mononuclear phagocyte system, play a crucial role in pathogen recognition and clearance and are important targets for RA treatment (33, 34). HLA DR on CD14- CD16+ monocytes, a subset of non-classical monocytes, is involved in promoting the resolution or disease progression in chronic inflammation (35). The role of non-classical monocytes in arthritis remains controversial. While Serum Transfer-Induced Arthritis (STIA) models show that non-classical monocytes are not essential for arthritis development and may even alleviate it, other studies have shown a positive correlation between non-classical monocytes and markers of joint destruction (36, 37). Previous research has indicated that there are fewer nonclassical monocytes in patients with RA than in the general population (38), but recent studies have shown an increase in these cells in the peripheral blood of patients with RA (39–41), which is consistent with our findings. This suggests the transformation of non-classical monocytes to classical monocytes in patients with RA. Analysis of synovial tissue biopsies from patients with RA revealed the accumulation of CD4+ CD8dim T cells in the synovial tissue (42). Our results revealed a negative correlation between RA and the expression of CD4+ CD8dim T cells as a percentage of lymphocytes and leukocytes, possibly suggesting that these cells are recruited from circulating peripheral blood to the joint fluid and synovium in patients with RA. There are no current studies on these cell levels in patients with RA, warranting further investigation. Previous studies have debated the B cell subtypes in RA, with some indicating a lower frequency of memory B cells in patients with RA than in healthy individuals, while others found no difference (43). Naïve mature B cells differentiate into memory B cells upon antigen contact and further differentiate into plasma cells through somatic hypermutation, class switch recombination, and other mechanisms resulting in antibody secretion (44). Our results revealed a negative correlation between RA and percentage of memory B cells among lymphocytes, suggesting the accumulation of memory B cells in the synovial tissue of patients with RA. The significant increase in circulating memory B cells after infliximab treatment in patients with RA further supports the idea of memory B cell migration to inflamed tissue, which can be corrected by tumor necrosis factor blockade (45). The decrease in B cells in patients with RA compared with healthy individuals may be due to the migration of B lymphocytes from peripheral blood to joint fluid, which promotes local inflammation through cytokine, chemokine secretion, and antigen presentation (43). This finding is consistent with our reverse MR results, indicating a negative correlation between RA and three B cell types (Memory B cell %lymphocyte, CD24 on IgD+ CD24+ B cell, and CD24 on unswitched memory B cell).

It has been demonstrated that higher levels of docosatrienoate (22:3n3) are associated with a reduced risk of RA. Docosatrienoate (22:3n-3), an omega-3 unsaturated fatty acid with 22 carbon atoms and 3 double bonds, has not been extensively studied clinically. Omega-3 fatty acids have been found to have anti-inflammatory and immunomodulatory effects through various mechanisms, such as altering the composition of fatty acids in cell membrane phospholipids and inhibiting the activation of pro-inflammatory factor kappa B, thereby suppressing the expression of inflammatory genes (46). Clinical studies have shown that omega-3, as an anti-inflammatory nutrient, has a positive effect on symptoms, cardiovascular events, and the progression of rheumatoid disease in patients with RA (46–48). Our findings provide genetic evidence for future research on the metabolic mechanisms of docosatrienoate (22:3n3) in RA.

Firstly, this study is the first to use the latest large-scale GWAS cohorts and multiple MR analysis methods to explore the causal relationships between immune cells, metabolites, and RA, using a series of sensitivity analyses to ensure the robustness of our results. Secondly, our study strictly followed the STROBE-MR guidelines and conclusions were based on MR-Egger regression horizontal pleiotropy negativity and FDR correction. However, while acknowledging the strengths of this study, its limitations must also be considered. First, to avoid population bias, all the groups involved in the study were of European ethnicity, which may affect the generalization of the conclusions. Second, due to the lack of personal information in the source data, this study was unable to stratify the analysis according to factors such as sex and age. Lastly, given the exploratory nature of our study, subsequent related studies based on our findings could introduce techniques such as single-cell RNA sequencing and fine-tuned localization.

## Conclusion

Bidirectional MR and mediation analyses were utilized to provide genetic evidence for elucidating the complex relationships between immune cells, plasma metabolites, and RA. This study provided genetic evidence for future researchers and clinicians to explore the prevention and treatment of RA.

## Data Availability

The datasets presented in this study can be found in online repositories. The names of the repository/repositories and accession number(s) can be found in the article/**Supplementary Material**.

## References

[1] Hanlon MM Canavan M Barker BE Fearon U. Metabolites as drivers and targets in rheumatoid arthritis. Clin Exp Immunol. (2022) 208:167–80. doi: 10.1093/cei/uxab021 PMC9188347 35020864

[2] Aletaha D Smolen JS. Diagnosis and management of rheumatoid arthritis: A review. JAMA. (2018) 320:1360–72. doi: 10.1001/jama.2018.13103 30285183

[3] Almutairi K Nossent J Preen D Keen H Inderjeeth C. The global prevalence of rheumatoid arthritis: a meta-analysis based on a systematic review. Rheumatol Int. (2021) 41:863–77. doi: 10.1007/s00296-020-04731-0 33175207

[4] Lundkvist J Kastäng F Kobelt G. The burden of rheumatoid arthritis and access to treatment: health burden and costs. Eur J Health Econ HEPAC Health Econ Prev Care. (2008) 8 Suppl 2:S49–60. doi: 10.1007/s10198-007-0088-8 18157732

[5] Finckh A Gilbert B Hodkinson B Bae SC Thomas R Deane KD. Global epidemiology of rheumatoid arthritis. Nat Rev Rheumatol. (2022) 18:591–602. doi: 10.1038/s41584-022-00827-y 36068354

[6] Jang S Kwon EJ Lee JJ. Rheumatoid arthritis: pathogenic roles of diverse immune cells. Int J Mol Sci. (2022) 23:905. doi: 10.3390/ijms23020905 35055087 PMC8780115

[7] Nerurkar L Siebert S McInnes IB Cavanagh J. Rheumatoid arthritis and depression: an inflammatory perspective. Lancet Psychiatry. (2019) 6:164–73. doi: 10.1016/S2215-0366(18)30255-4 30366684

[8] K N T H. Mechanisms of joint destruction in rheumatoid arthritis - immune cell-fibroblast-bone interactions. Nat Rev Rheumatol. (2022) 18:415–29. doi: 10.1038/s41584-022-00793-5. 35705856

[9] Zheng Y Liu Q Goronzy JJ Weyand CM. Immune aging - A mechanism in autoimmune disease. Semin Immunol. (2023) 69:101814. doi: 10.1016/j.smim.2023.101814 37542986 PMC10663095

[10] Johnson CH Ivanisevic J Siuzdak G. Metabolomics: beyond biomarkers and towards mechanisms. Nat Rev Mol Cell Biol. (2016) 17:451–9. doi: 10.1038/nrm.2016.25 PMC5729912 26979502

[11] Wishart DS. Metabolomics for investigating physiological and pathophysiological processes. Physiol Rev. (2019) 99:1819–75. doi: 10.1152/physrev.00035.2018 31434538

[12] Coras R Murillo-Saich JD Guma M. Circulating pro- and anti-inflammatory metabolites and its potential role in rheumatoid arthritis pathogenesis. Cells. (2020) 9:827. doi: 10.3390/cells9040827 32235564 PMC7226773

[13] Xu L Chang C Jiang P Wei K Zhang R Jin Y. Metabolomics in rheumatoid arthritis: Advances and review. Front Immunol. (2022) 13:961708. doi: 10.3389/fimmu.2022.961708 36032122 PMC9404373

[14] Bowden J Holmes MV. Meta-analysis and Mendelian randomization: A review. Res Synth Methods. (2019) 10:486–96. doi: 10.1002/jrsm.1346 PMC6973275 30861319

[15] Skrivankova VW Richmond RC Woolf BAR Yarmolinsky J Davies NM Swanson SA. Strengthening the reporting of observational studies in epidemiology using Mendelian randomization: the STROBE-MR statement. JAMA. (2021) 326:1614–21. doi: 10.1001/jama.2021.18236 34698778

[16] Gagliano Taliun SA Evans DM. Ten simple rules for conducting a mendelian randomization study. PLoS Comput Biol. (2021) 17:e1009238. doi: 10.1371/journal.pcbi.1009238 34383747 PMC8360373

[17] Orrù V Steri M Sidore C Marongiu M Serra V Olla S. Complex genetic signatures in immune cells underlie autoimmunity and inform therapy. Nat Genet. (2020) 52:1036–45. doi: 10.1038/s41588-020-0684-4 PMC8517961 32929287

[18] Index of /pub/databases/gwas/summary_statistics. Available online at: https://ftp.ebi.ac.uk/pub/databases/gwas/summary_statistics/. (Accesed May 5, 2024).

[19] Chen Y Lu T Pettersson-Kymmer U Stewart ID Butler-Laporte G Nakanishi T. Genomic atlas of the plasma metabolome prioritizes metabolites implicated in human diseases. Nat Genet. (2023) 55:44–53. doi: 10.1038/s41588-022-01270-1 36635386 PMC7614162

[20] FinnGen. FinnGen: an expedition into genomics and medicine. Available online at: https://www.finngen.fi/en/finngen_research_project_is_an_expedition_to_the_frontier_of_genomics_and_medicine. (Accesed May 5, 2024).

[21] Kurki MI Karjalainen J Palta P Sipilä TP Kristiansson K Donner KM. FinnGen provides genetic insights from a well-phenotyped isolated population. Nature. (2023) 613:508–18. doi: 10.1038/s41586-022-05473-8 PMC9849126 36653562

[22] Gao B Wang Z Wang K Lei Y Zhuang Y Zhou Z. Relationships among gut microbiota, plasma metabolites, and juvenile idiopathic arthritis: a mediation Mendelian randomization study. Front Microbiol. (2024) 15:1363776. doi: 10.3389/fmicb.2024.1363776 38605717 PMC11007183

[23] Burgess S Thompson SG CRP CHD Genetics Collaboration. Avoiding bias from weak instruments in Mendelian randomization studies. Int J Epidemiol. (2011) 40:755–64. doi: 10.1093/ije/dyr036 21414999

[24] Korthauer K Kimes PK Duvallet C Reyes A Subramanian A Teng M. A practical guide to methods controlling false discoveries in computational biology. Genome Biol. (2019) 20:118. doi: 10.1186/s13059-019-1716-1 31164141 PMC6547503

[25] Bowden J Del Greco MF Minelli C Davey Smith G Sheehan NA Thompson JR. Assessing the suitability of summary data for two-sample Mendelian randomization analyses using MR-Egger regression: the role of the I2 statistic. Int J Epidemiol. (2016) 45:1961–74. doi: 10.1093/ije/dyw220 PMC5446088 27616674

[26] Hao X Ren C Zhou H Li M Zhang H Liu X. Association between circulating immune cells and the risk of prostate cancer: a Mendelian randomization study. Front Endocrinol. (2024) 15:1358416. doi: 10.3389/fendo.2024.1358416 PMC10884280 38405157

[27] Zouali M. DNA methylation signatures of autoimmune diseases in human B lymphocytes. Clin Immunol Orlando Fla. (2021) 222:108622. doi: 10.1016/j.clim.2020.108622 33188932

[28] Schett G Mackensen A Mougiakakos D. CAR T-cell therapy in autoimmune diseases. Lancet Lond Engl. (2023) 402:2034–44. doi: 10.1016/S0140-6736(23)01126-1 37748491

[29] Wu F Gao J Kang J Wang X Niu Q Liu J. B cells in rheumatoid arthritis: Pathogenic mechanisms and treatment prospects. Front Immunol. (2021) 12:750753. doi: 10.3389/fimmu.2021.750753 34650569 PMC8505880

[30] Roudier J Balandraud N Auger I. How RA associated HLA-DR molecules contribute to the development of antibodies to citrullinated proteins: the hapten carrier model. Front Immunol. (2022) 13:930112. doi: 10.3389/fimmu.2022.930112 35774784 PMC9238433

[31] Wysocki T Olesińska M Paradowska-Gorycka A. Current understanding of an emerging role of HLA-DRB1 gene in rheumatoid arthritis-from research to clinical practice. Cells. (2020) 9:1127. doi: 10.3390/cells9051127 32370106 PMC7291248

[32] Ting YT Petersen J Ramarathinam SH Scally SW Loh KL Thomas R. The interplay between citrullination and HLA-DRB1 polymorphism in shaping peptide binding hierarchies in rheumatoid arthritis. J Biol Chem. (2018) 293:3236–51. doi: 10.1074/jbc.RA117.001013 PMC5836122 29317506

[33] Smolen JS Aletaha D Barton A Burmester GR Emery P Firestein GS. Rheumatoid arthritis. Nat Rev Dis Primer. (2018) 4:18002. doi: 10.1038/nrdp.2018.2 29417936

[34] H Mm M T M V A S S Q N S. Rheumatoid arthritis macrophages are primed for inflammation and display bioenergetic and functional alterations. Rheumatol Oxf Engl. (2023) 62:2611–20. doi: 10.1093/rheumatology/keac640. PMC10321118 36398893

[35] Narasimhan PB Marcovecchio P Hamers AAJ Hedrick CC. Nonclassical monocytes in health and disease. Annu Rev Immunol. (2019) 37:439–56. doi: 10.1146/annurev-immunol-042617-053119 31026415

[36] Brunet A LeBel M Egarnes B Paquet-Bouchard C Lessard AJ Brown JP. NR4A1-dependent Ly6Clow monocytes contribute to reducing joint inflammation in arthritic mice through Treg cells. Eur J Immunol. (2016) 46:2789–800. doi: 10.1002/eji.201646406 27600773

[37] Puchner A Saferding V Bonelli M Mikami Y Hofmann M Brunner JS. Non-classical monocytes as mediators of tissue destruction in arthritis. Ann Rheum Dis. (2018) 77:1490–7. doi: 10.1136/annrheumdis-2018-213250 PMC6161666 29959183

[38] Klimek E Mikołajczyk T Sulicka J Kwaśny-Krochin B Korkosz M Osmenda G. Blood monocyte subsets and selected cardiovascular risk markers in rheumatoid arthritis of short duration in relation to disease activity. BioMed Res Int. (2014) 2014:736853. doi: 10.1155/2014/736853 25126574 PMC4122153

[39] Lacerte P Brunet A Egarnes B Duchêne B Brown JP Gosselin J. Overexpression of TLR2 and TLR9 on monocyte subsets of active rheumatoid arthritis patients contributes to enhance responsiveness to TLR agonists. Arthritis Res Ther. (2016) 18:10. doi: 10.1186/s13075-015-0901-1 26759164 PMC4718023

[40] Smiljanovic B Radzikowska A Kuca-Warnawin E Kurowska W Grün JR Stuhlmüller B. Monocyte alterations in rheumatoid arthritis are dominated by preterm release from bone marrow and prominent triggering in the joint. Ann Rheum Dis. (2018) 77:300–8. doi: 10.1136/annrheumdis-2017-211649 PMC5867420 29191820

[41] Prajzlerová K Kryštůfková O Komarc M Mann H Hulejová H Petrovská N. The dysregulation of monocyte subpopulations in individuals at risk of developing rheumatoid arthritis. Rheumatol Oxf Engl. (2021) 60:1823–31. doi: 10.1093/rheumatology/keaa518 33119082

[42] Floudas A Neto N Orr C Canavan M Gallagher P Hurson C. Loss of balance between protective and pro-inflammatory synovial tissue T-cell polyfunctionality predates clinical onset of rheumatoid arthritis. Ann Rheum Dis. (2022) 81:193–205. doi: 10.1136/annrheumdis-2021-220458 34598926

[43] Pala O Diaz A Blomberg BB Frasca D. B lymphocytes in rheumatoid arthritis and the effects of anti-TNF-α Agents on B lymphocytes: A review of the literature. Clin Ther. (2018) 40:1034–45. doi: 10.1016/j.clinthera.2018.04.016 PMC6792291 29801753

[44] Wu H Deng Y Feng Y Long D Ma K Wang X. Epigenetic regulation in B-cell maturation and its dysregulation in autoimmunity. Cell Mol Immunol. (2018) 15:676–84. doi: 10.1038/cmi.2017.133 PMC6123482 29375128

[45] Souto-Carneiro MM Mahadevan V Takada K Fritsch-Stork R Nanki T Brown M. Alterations in peripheral blood memory B cells in patients with active rheumatoid arthritis are dependent on the action of tumor necrosis factor. Arthritis Res Ther. (2009) 11:R84. doi: 10.1186/ar2718 19500335 PMC2714135

[46] Fares S Omar M Laurence A Abu-Baker S Shaza A Fadi H. Over-the-counter anti-inflammatory supplements for adjunctive rheumatoid arthritis therapy: A comprehensive narrative review. Aging Dis. (2024). doi: 10.14336/AD.2024.0131 PMC11745436 38377032

[47] Calder PC. Omega-3 fatty acids and inflammatory processes: from molecules to man. Biochem Soc Trans. (2017) 45:1105–15. doi: 10.1042/BST20160474 28900017

[48] Tomic-Smiljanic M Vasiljevic D Lucic-Tomic A Andjelkovic N Jakovljevic V Bolovich S. Influence of different supplementation on platelet aggregation in patients with rheumatoid arthritis. Clin Rheumatol. (2019) 38:2443–50. doi: 10.1007/s10067-019-04569-3 31076942

