# Supplementary material for: Effect of the immune cells and plasma metabolites on rheumatoid arthritis: a mediated mendelian randomization study
Source: Front Endocrinol (Lausanne). 2024 Sep 3;15:1438097. doi: 10.3389/fendo.2024.1438097 (PMC11407113; doi:10.3389/fendo.2024.1438097)
Supplement: Supplementary file 2 [file DataSheet2.zip › Data Sheet 2/Supplementary Figure Abstract.docx]

Effect of the Immune Cells and Plasma Metabolites on Rheumatoid Arthritis: A Mediated Mendelian Randomization Study

Qi-Pei Liu^12^ Hong-Cheng Du^3^ Ping-Jin Xie^4^ Sheng-Ting Chai^5*^

1 The Third School of Clinical Medicine, Guangzhou University of Chinese Medicine, Guangzhou, China

2 Graduate School of Guangzhou University of Chinese Medicine, Guangzhou, China

3 Graduate School of Guangxi University of Chinese Medicine, Nanning, China

4 Shenzhen Hospital of Shanghai University of Traditional Chinese Medicine, Shenzhen, China

5* Address for correspondence Sheng-Ting Chai, MD, PhD, Department of Arthrosis, the Third Affiliated Hospital of Guangzhou University of Chinese Medicine, Guangzhou, 510378, China (e-mail: [cst0192@qq.com](mailto:cst0192@qq.com)).

**Supplementary Figure captions:**

**Supplementary Figure S1.** Forest plot of Mendelian Randomization analysis between immune cell phenotypes and rheumatoid arthritis, meeting both inverse-variance weighted significance and MR-Egger regression pleiotropy neutrality.

**Supplementary Figure S2.** Summary forest plot of positive causal associations between immune cell phenotypes and rheumatoid arthritis after false discovery rate correction.

**Supplementary Figure S3.** Summary scatter plot of positive causal associations between immune cell phenotypes and rheumatoid arthritis after false discovery rate correction.

**Supplementary Figure S4.** Summary leave-one-out analysis of positive causal associations between immune cell phenotypes and rheumatoid arthritis after false discovery rate correction.

**Supplementary Figure S5.** Summary funnel plot of positive causal associations between immune cell phenotypes and rheumatoid arthritis after false discovery rate correction.

**Supplementary Figure S6.** Forest plot of Mendelian randomization analysis between rheumatoid arthritis and immune cell phenotypes, meeting both inverse-variance weighted significance and MR-Egger regression pleiotropy neutrality.

**Supplementary Figure S7.** Summary forest plot of positive causal associations between rheumatoid arthritis and immune cell phenotypes after false discovery rate correction.

**Supplementary Figure S8.** Summary scatter plot of positive causal associations between rheumatoid arthritis and immune cell phenotypes after false discovery rate correction.

**Supplementary Figure S9.** Summary leave-one-out analysis of positive causal associations between immune cell phenotypes and rheumatoid arthritis after false discovery rate correction.

**Supplementary Figure S10.** Summary funnel plot of positive causal associations between immune cell phenotypes and rheumatoid arthritis after false discovery rate correction.

**Supplementary Figure S11.** Forest plot of Mendelian randomization analysis between plasma metabolites and rheumatoid arthritis, meeting both inverse-variance weighted significance and MR-Egger regression pleiotropy neutrality.

**Supplementary Figure S12.** Summary of forest plots, scatter plots, leave-one-out analyses, and funnel plots of positive causal associations between plasma metabolites and rheumatoid arthritis after false discovery rate correction.

**Supplementary Figure S13.** Summary of forest plots, scatter plots, leave-one-out analyses, and funnel plots of positive causal associations between rheumatoid arthritis and plasma metabolites.

**Supplementary Figure S14.** Summary of forest plots, scatter plots, leave-one-out analyses, and funnel plots of causal associations between CD25 on IgD+ CD24+ and docosatrienoate (22:3n3) levels.

**Supplementary Figure S15.** Summary of forest plots, scatter plots, leave-one-out analyses, and funnel plots of causal associations between CD25 on IgD+ CD24+ and rheumatoid arthritis.

**Supplementary Figure S16.** Summary of forest plots, scatter plots, leave-one-out analyses, and funnel plots of causal associations between docosatrienoate (22:3n3) levels and rheumatoid arthritis.


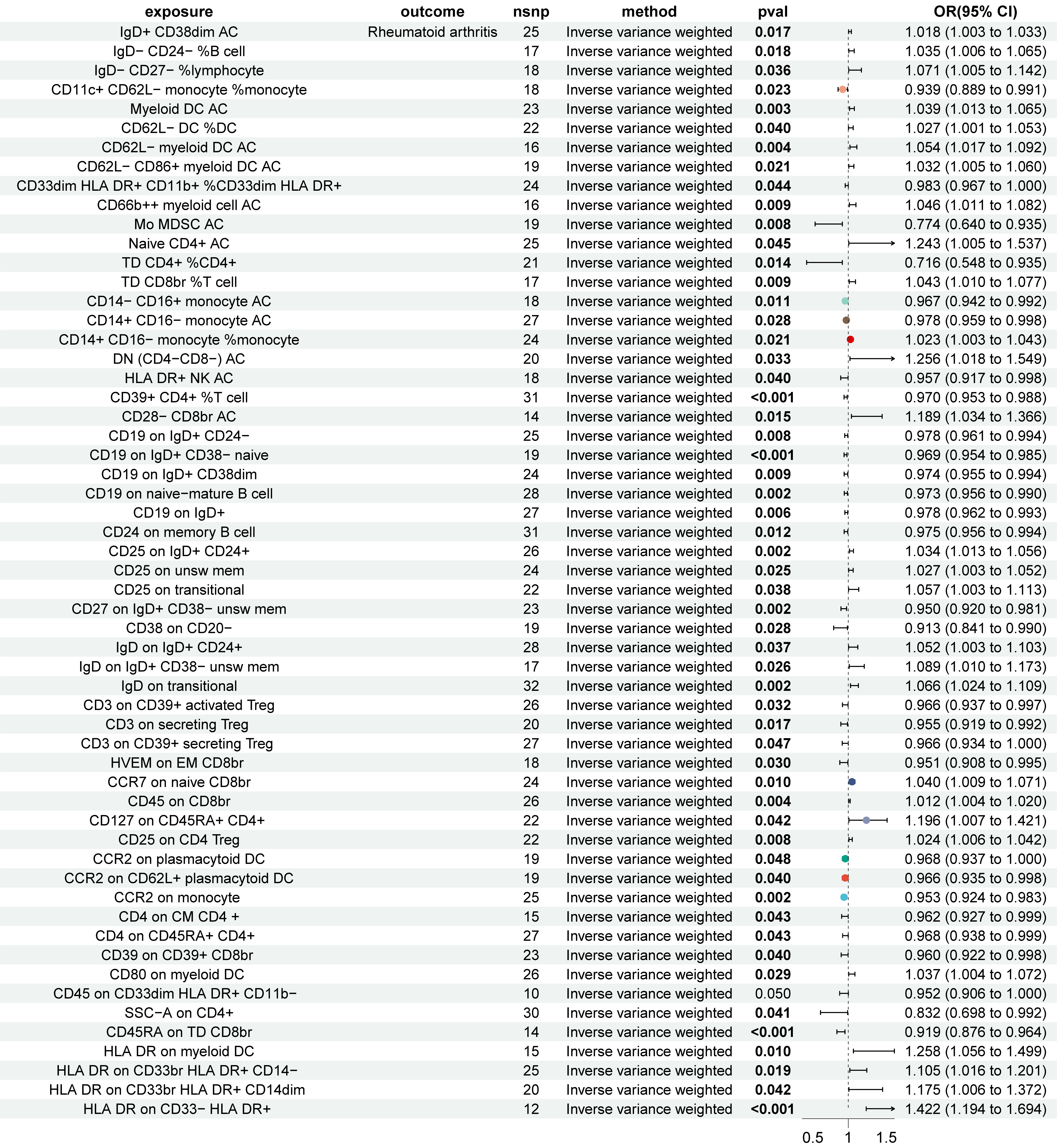


**Supplementary Figure S1.** Forest plot of Mendelian randomization analysis between immune cell phenotypes and rheumatoid arthritis, meeting both inverse-variance weighted significance and MR-Egger regression pleiotropy neutrality.


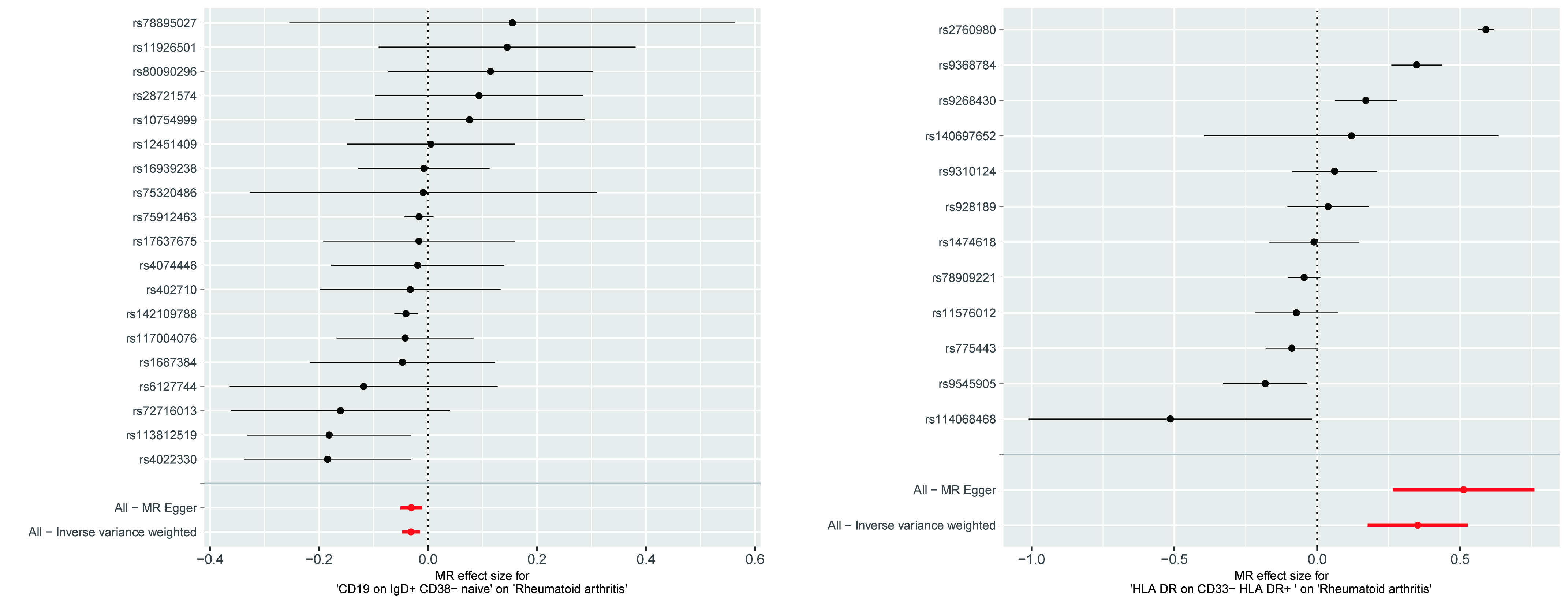


**Supplementary Figure S2.** Summary forest plot of positive causal associations between immune cell phenotypes and rheumatoid arthritis after false discovery rate correction.


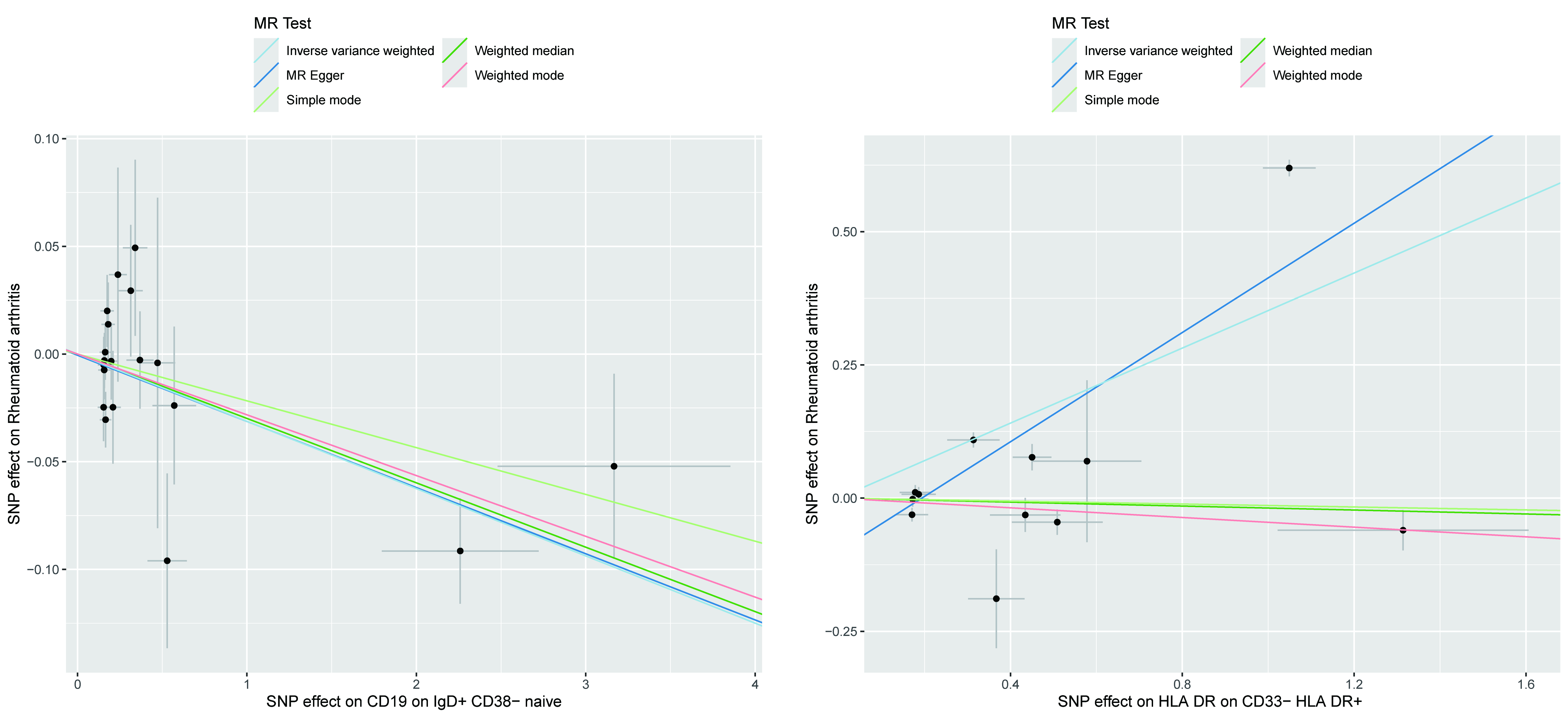


**Supplementary Figure S3.** Summary scatter plot of positive causal associations between immune cell phenotypes and rheumatoid arthritis after false discovery rate correction.


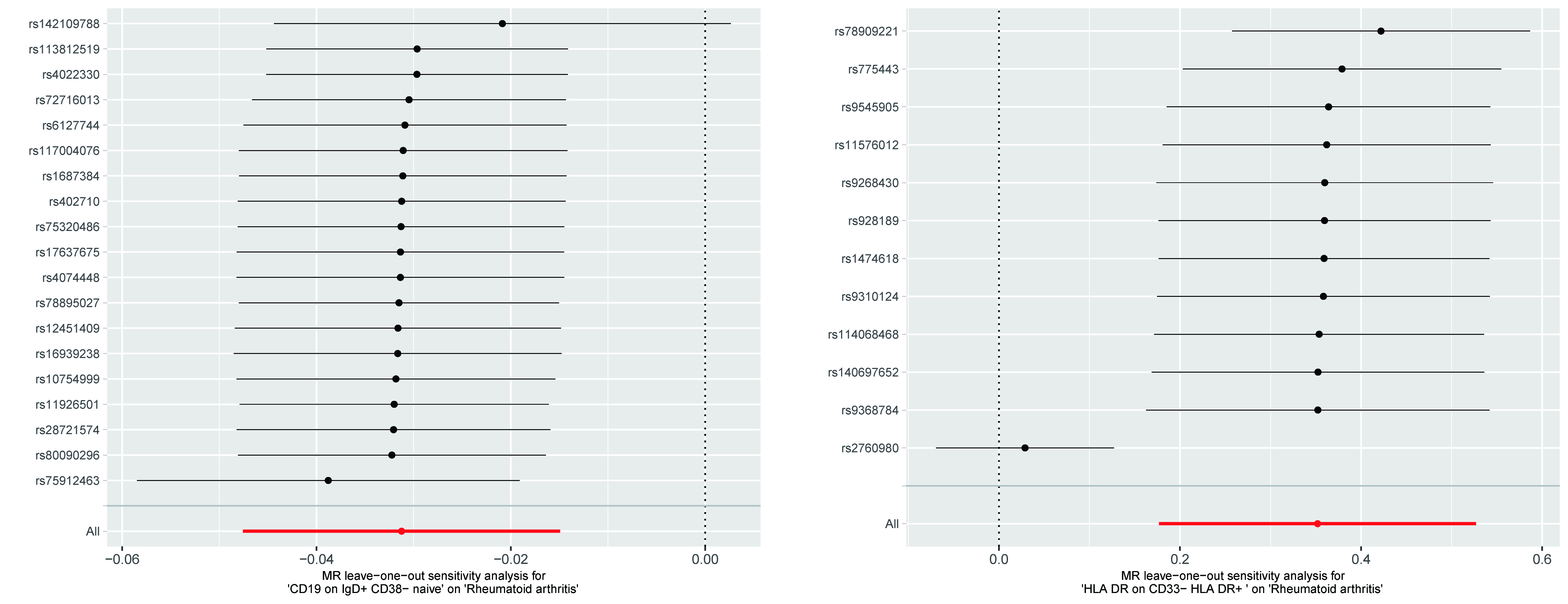


**Supplementary Figure S4.** Summary leave-one-out analysis of positive causal associations between immune cell phenotypes and rheumatoid arthritis after false discovery rate correction.


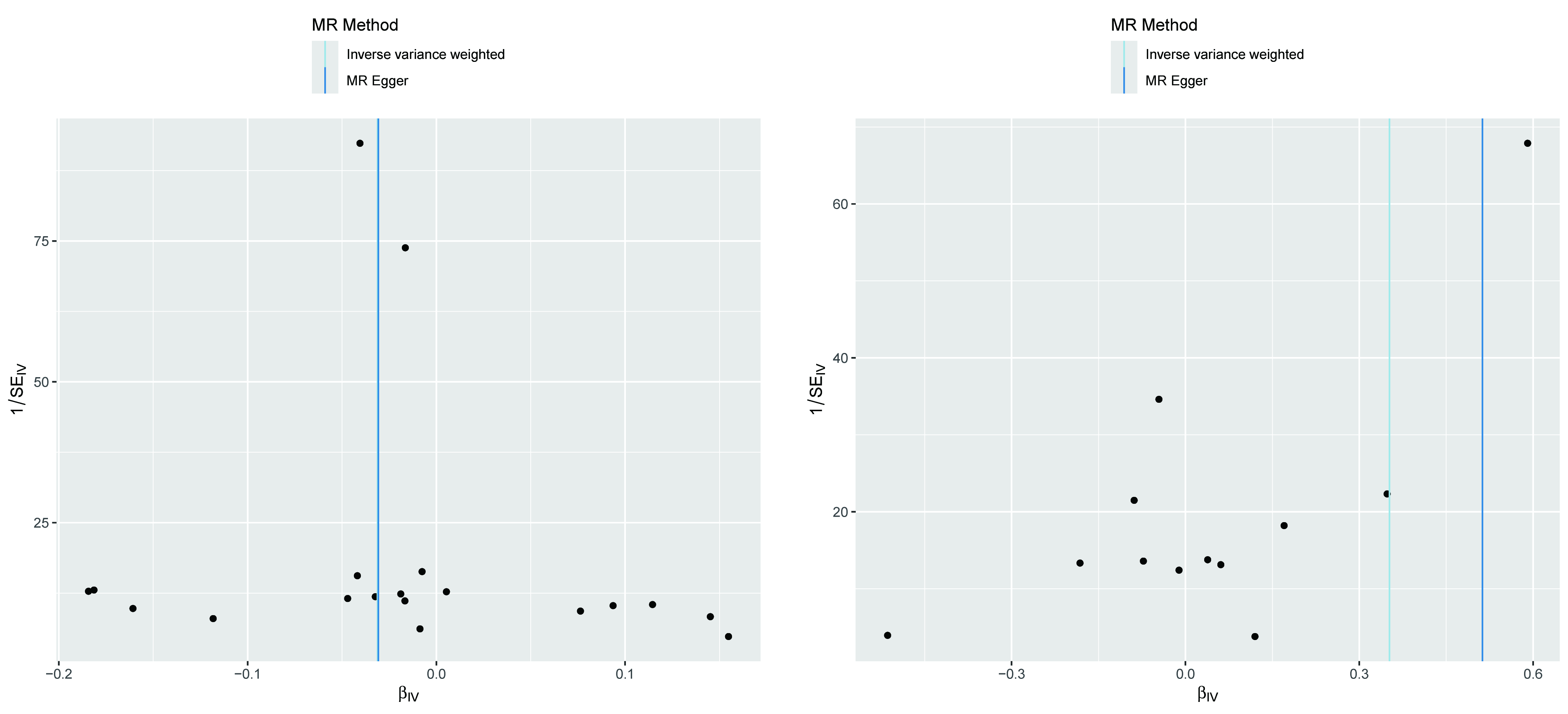


**Supplementary Figure S5.** Summary funnel plot of positive causal associations between immune cell phenotypes and rheumatoid arthritis after false discovery rate correction.

**
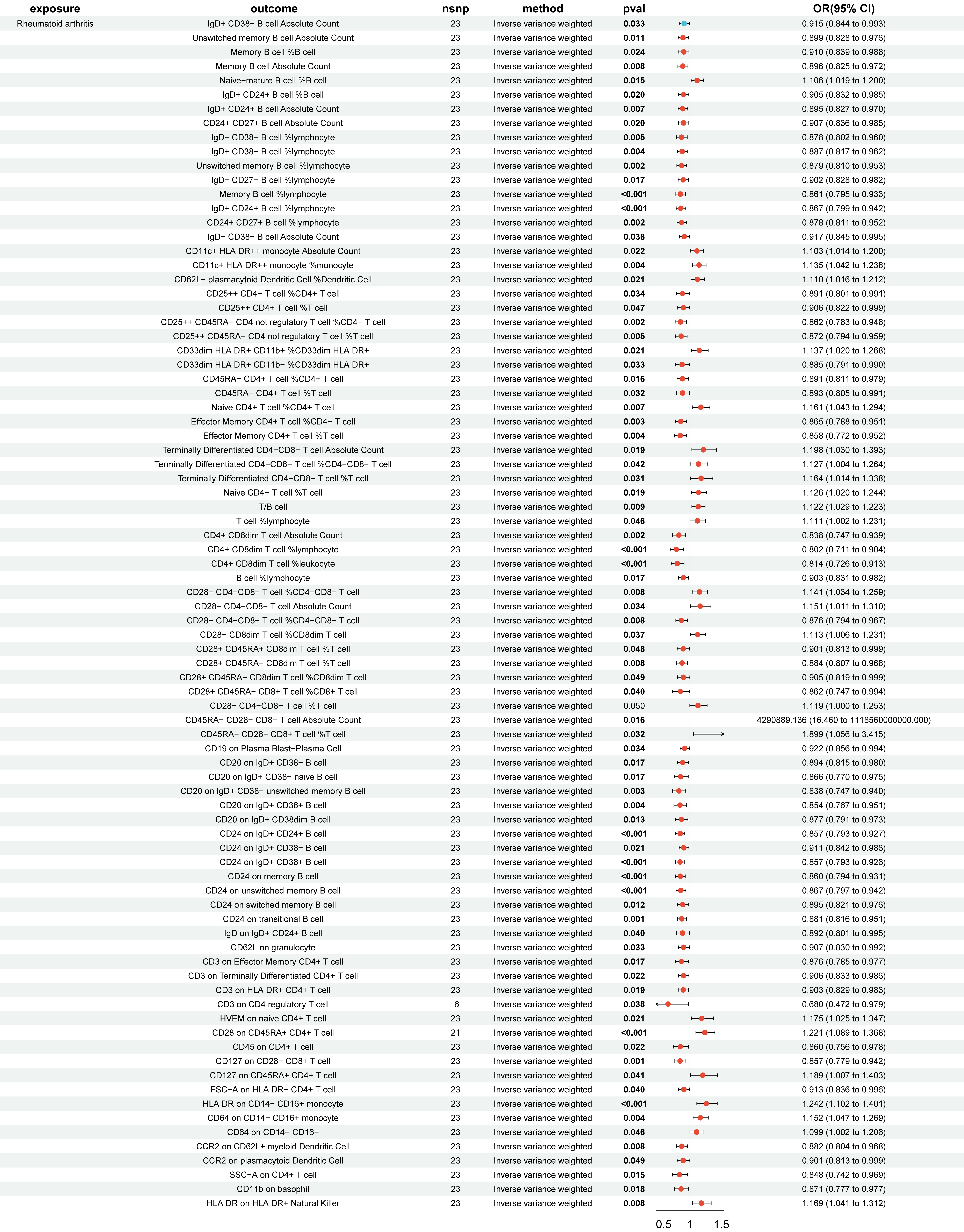
 Supplementary Figure S6.** Forest plot of Mendelian randomization analysis between rheumatoid arthritis and immune cell phenotypes, meeting both inverse-variance weighted significance and MR-Egger regression pleiotropy neutrality.

**
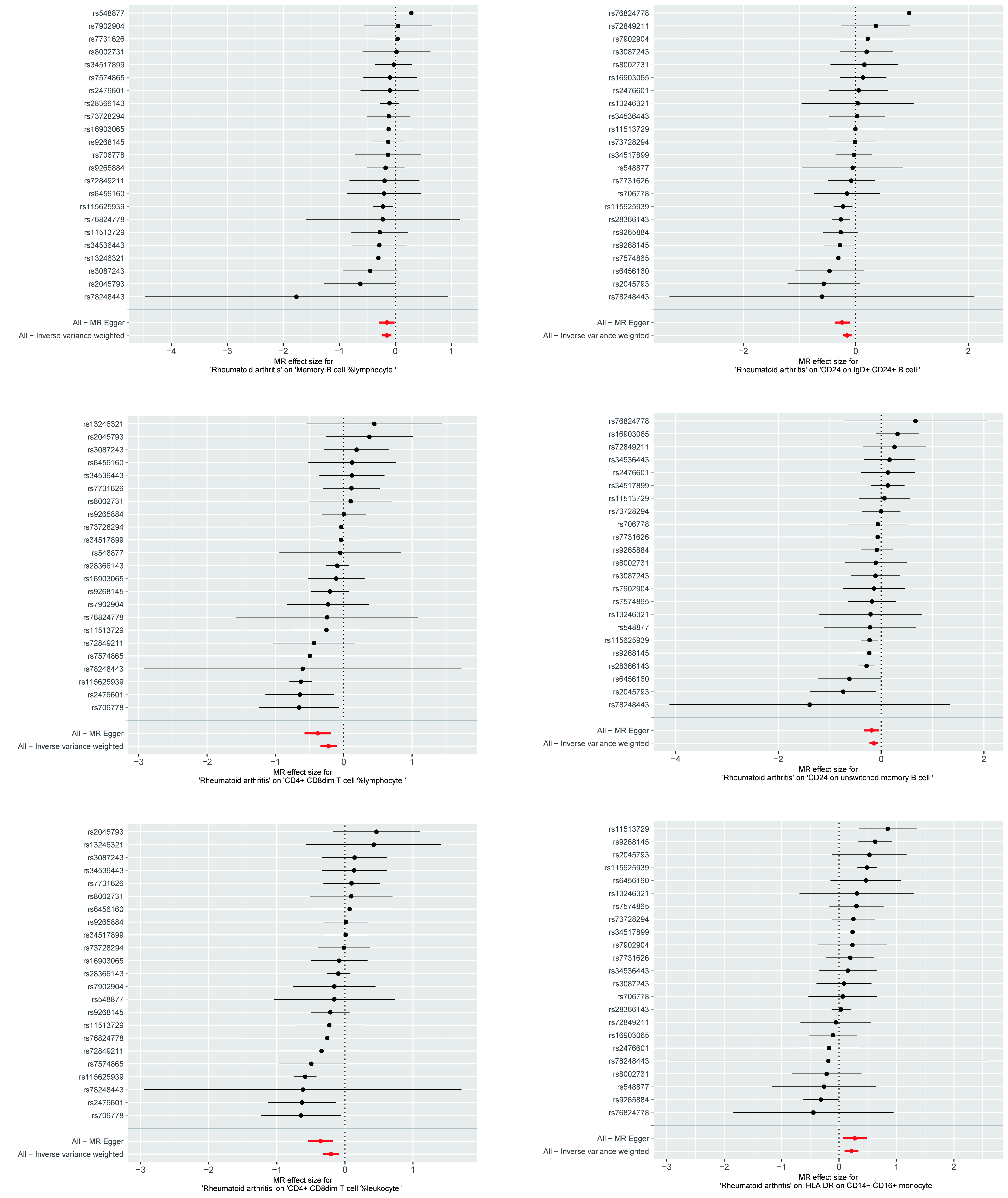
 Supplementary Figure S7.** Summary forest plot of positive causal associations between rheumatoid arthritis and immune cell phenotypes after false discovery rate correction.





**Supplementary Figure S8.** Summary scatter plot of positive causal associations between rheumatoid arthritis and immune cell phenotypes after false discovery rate correction. **
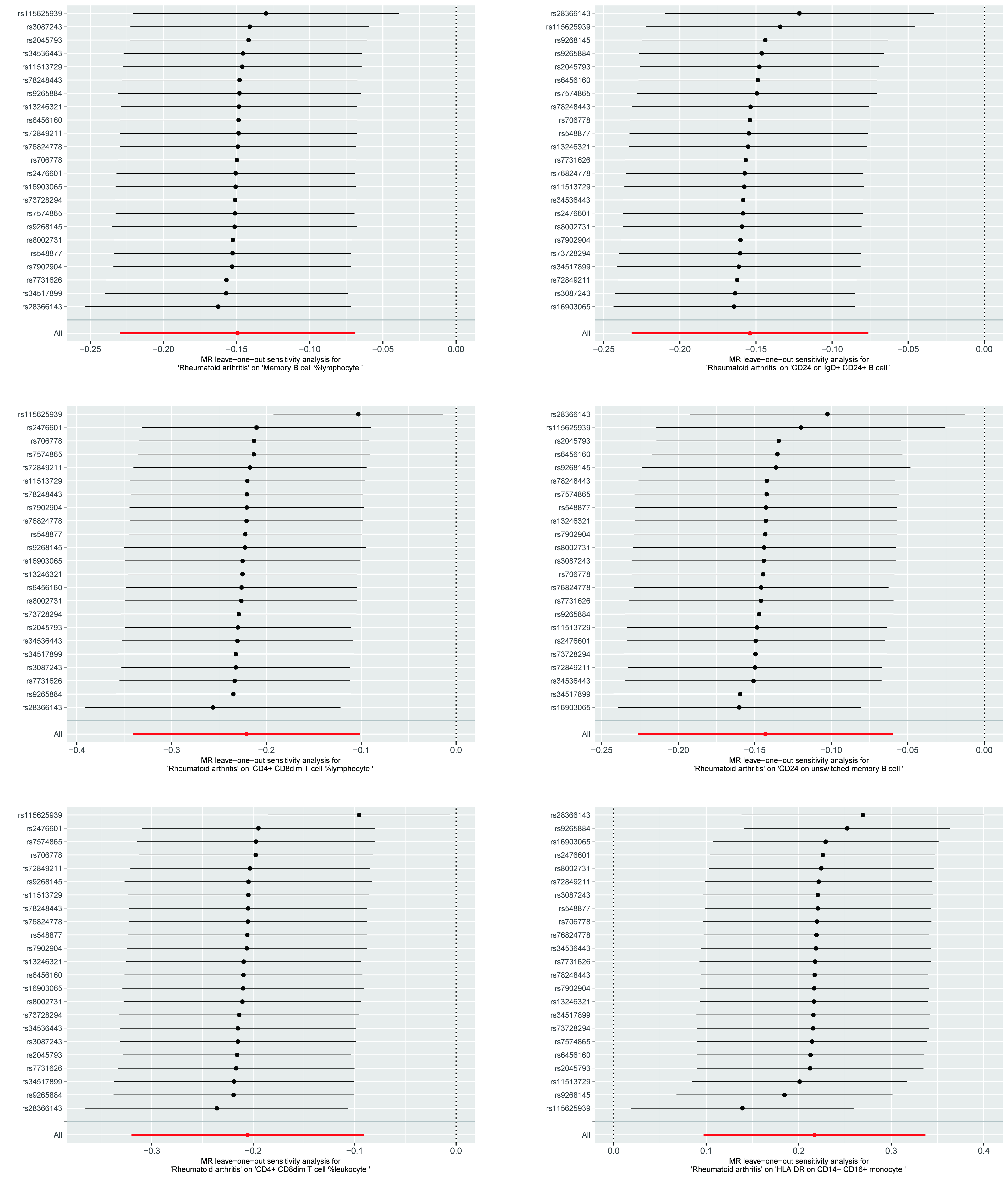
 Supplementary Figure S9.** Summary leave-one-out analysis of positive causal associations between immune cell phenotypes and rheumatoid arthritis after false discovery rate correction.

**
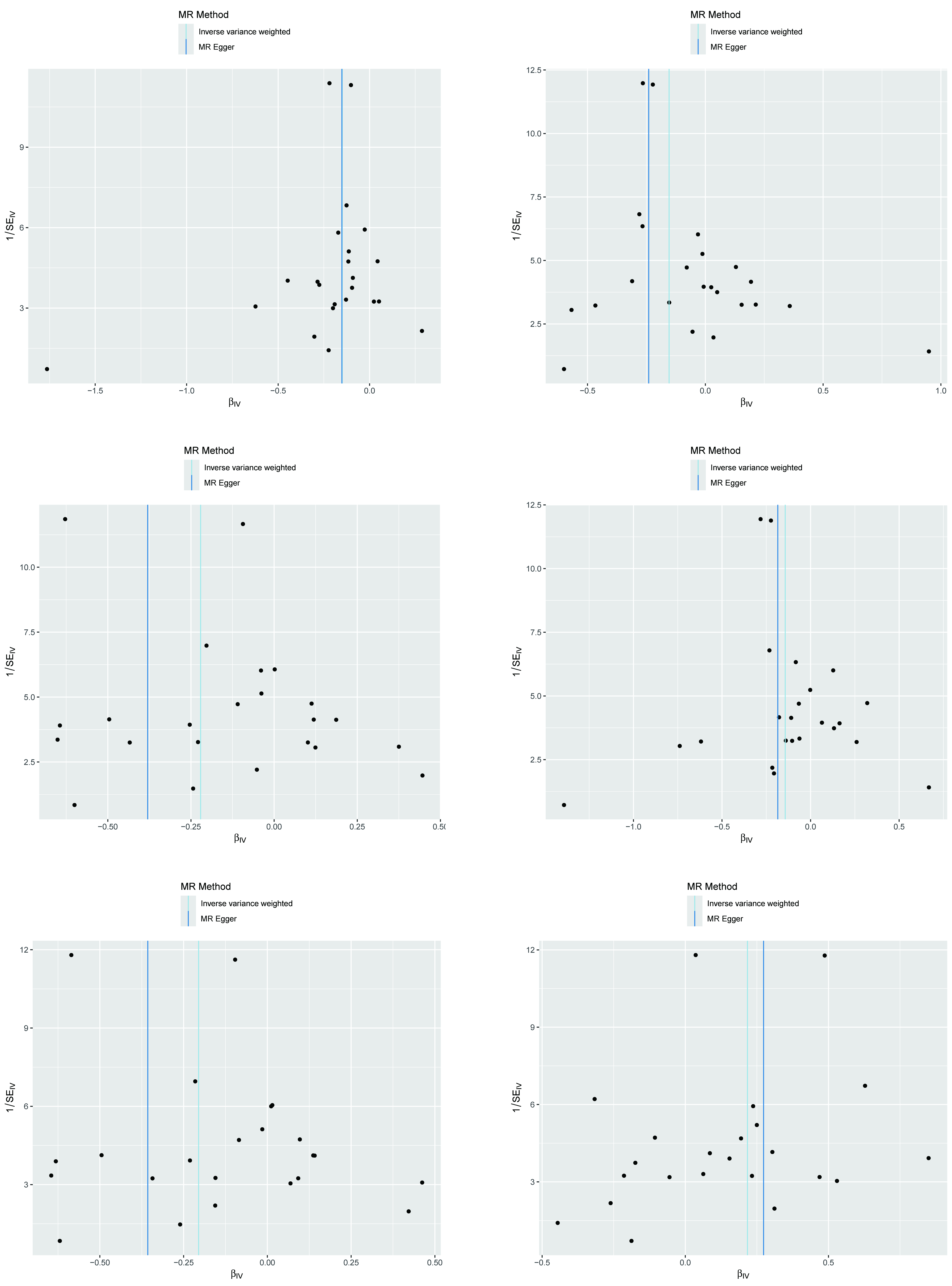
 Supplementary Figure S10.** Summary funnel plot of positive causal associations between immune cell phenotypes and rheumatoid arthritis after false discovery rate correction.

**
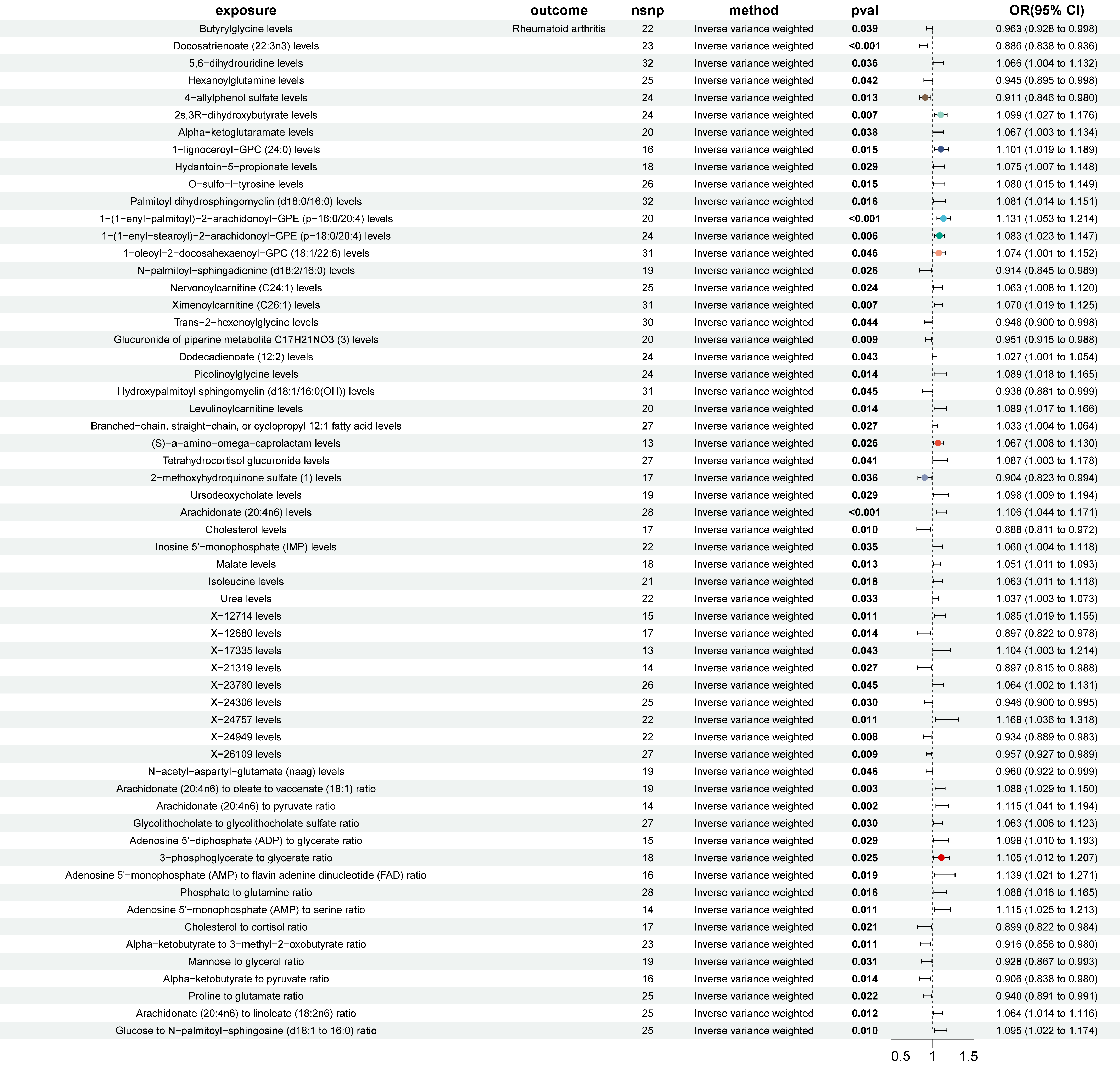
Supplementary Figure S11.** Forest plot of Mendelian randomization analysis between plasma metabolites and rheumatoid arthritis, meeting both inverse variance-weighted significance and MR-Egger regression pleiotropy neutrality.


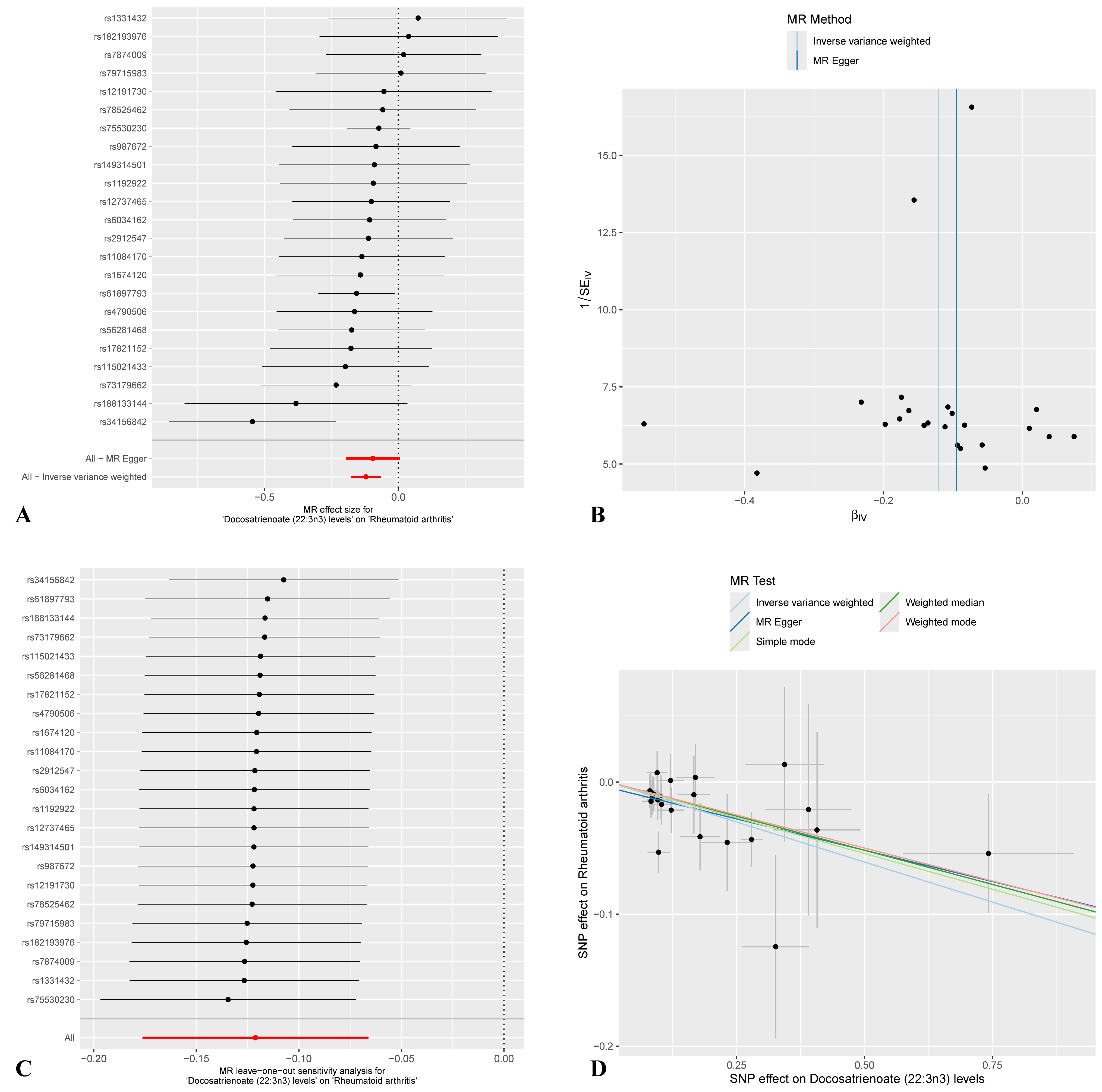


**Supplementary Figure S12.** Summary of forest plots, scatter plots, leave-one-out analyses, and funnel plots of positive causal associations between plasma metabolites and rheumatoid arthritis after false discovery rate correction.
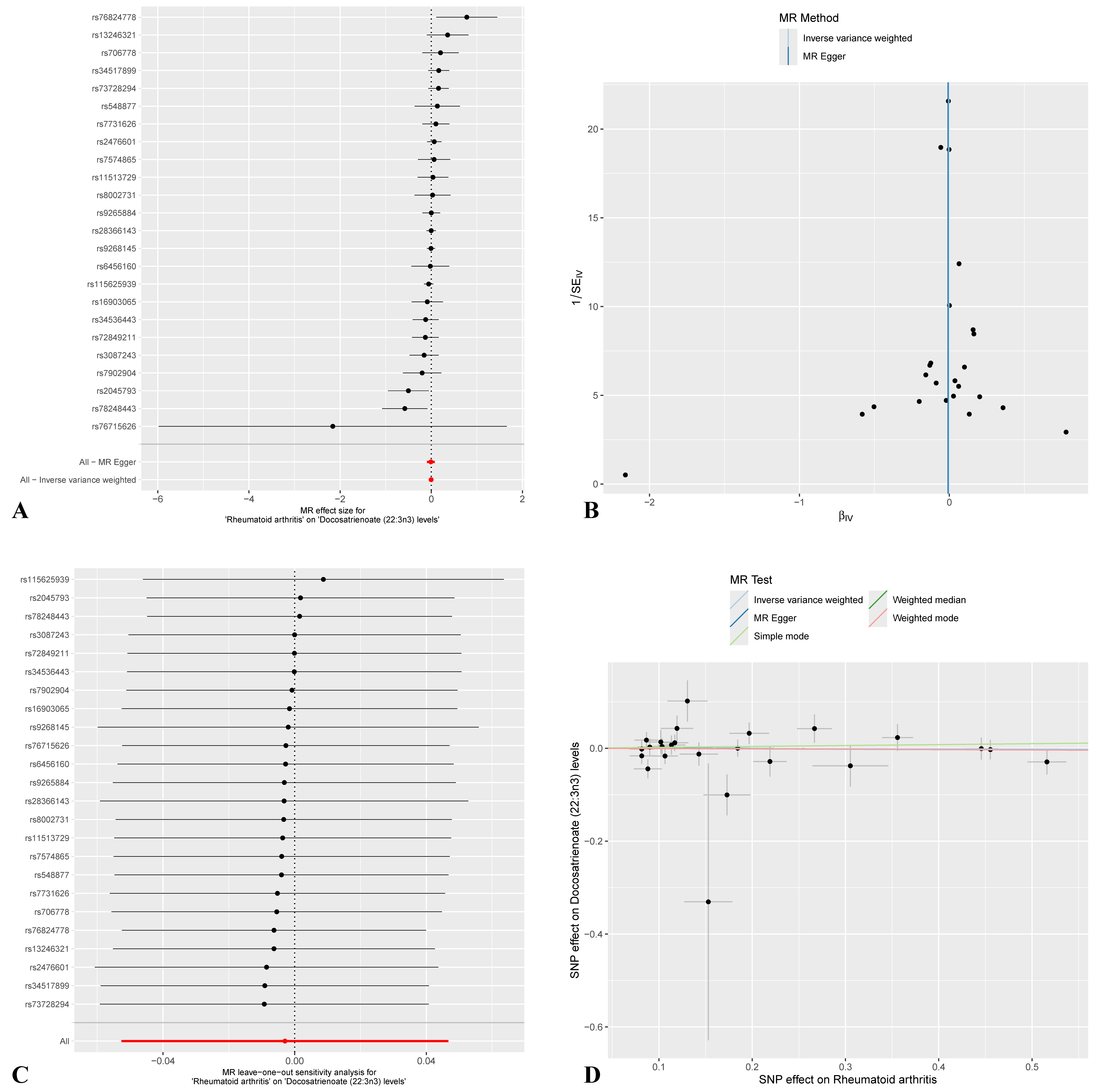


**Supplementary Figure S13.** Summary of forest plots, scatter plots, leave-one-out analyses, and funnel plots of positive causal associations between rheumatoid arthritis and plasma metabolites.
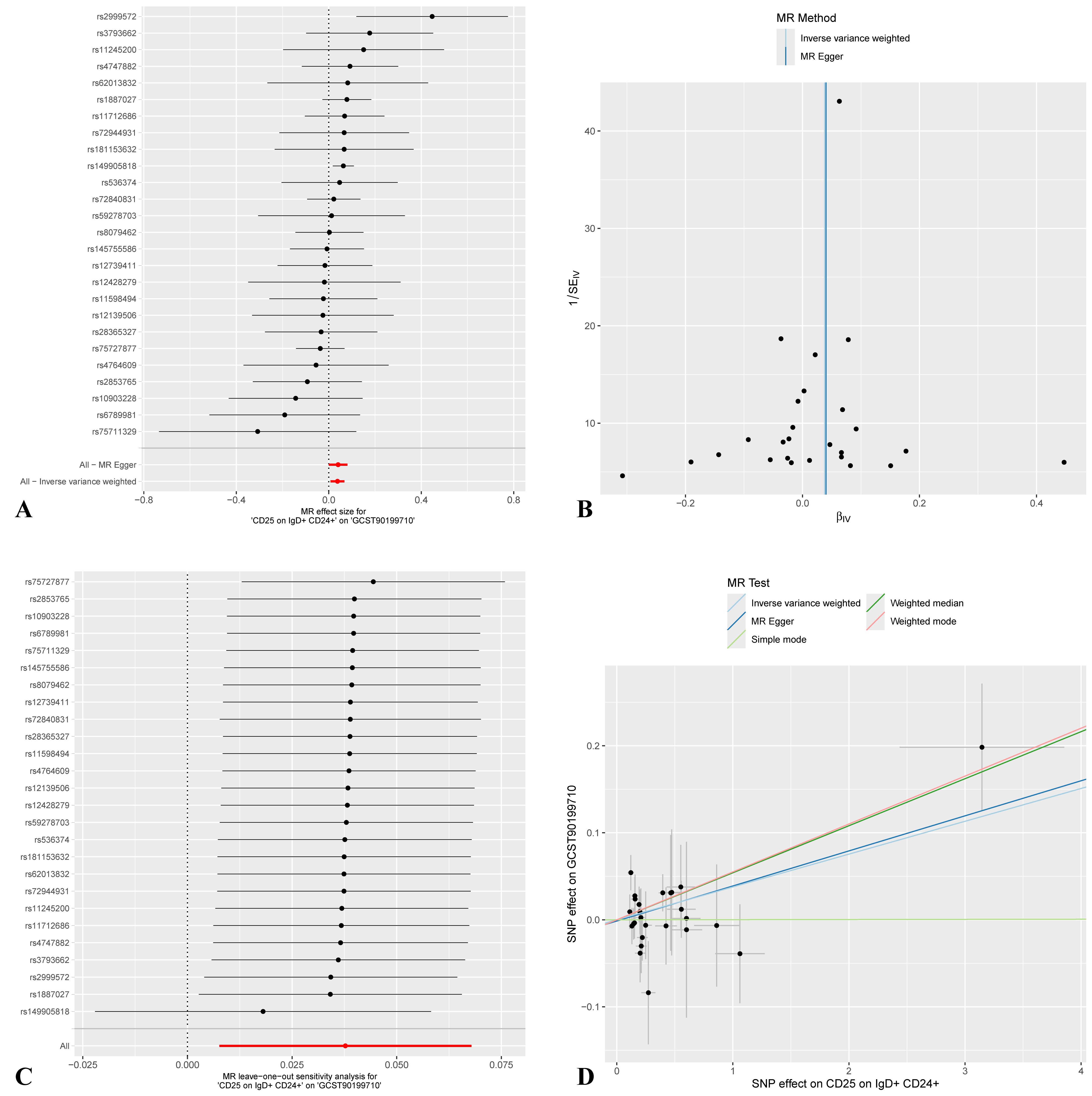


**Supplementary Figure S14.** Summary of forest plots, scatter plots, leave-one-out analyses, and funnel plots of causal associations between CD25 on IgD+ CD24+ and docosatrienoate (22:3n3) levels.
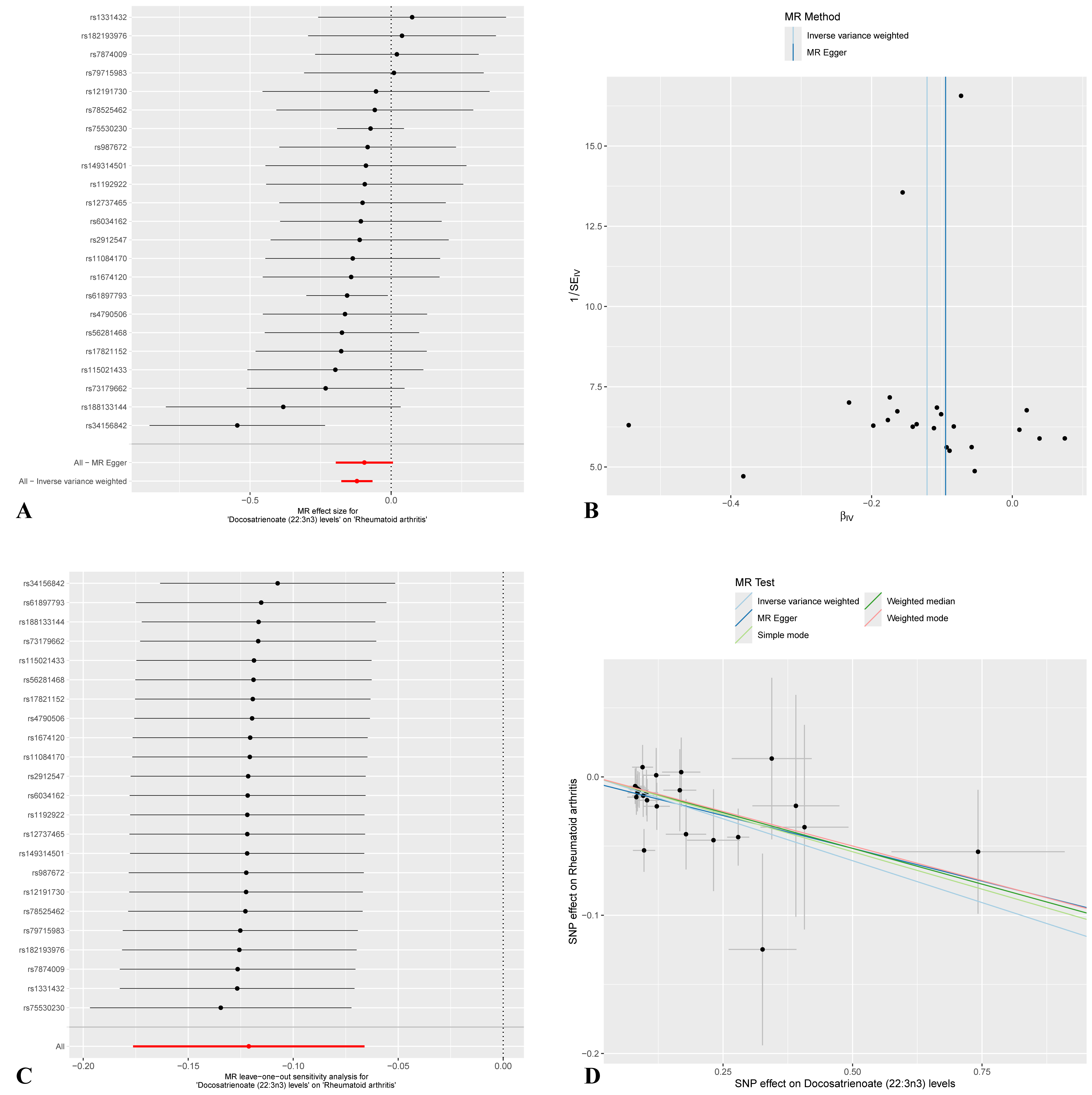


**Supplementary Figure S15.** Summary of forest plots, scatter plots, leave-one-out analyses, and funnel plots of causal associations between CD25 on IgD+ CD24+ and rheumatoid arthritis.
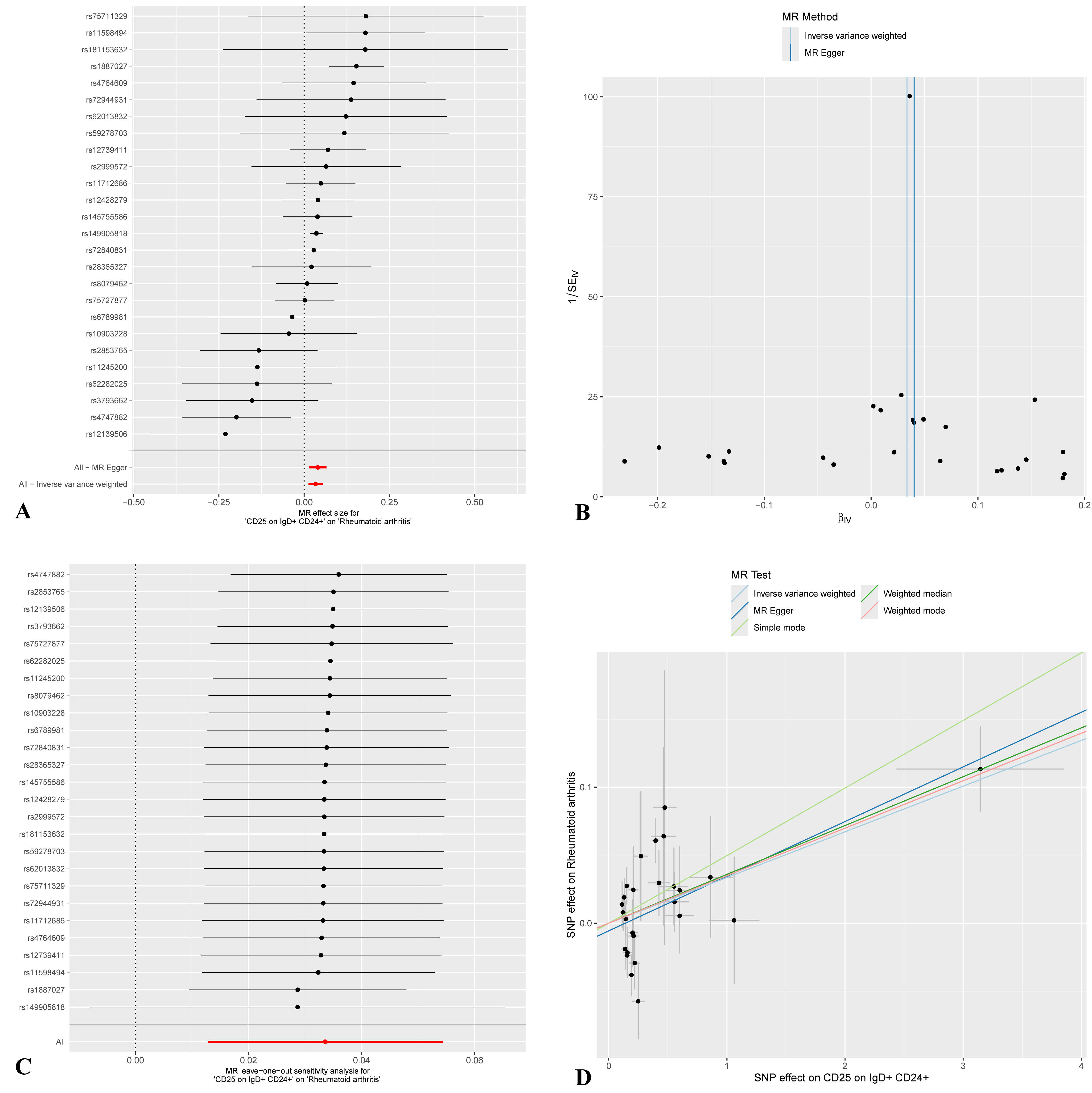


**Supplementary Figure S16.** Summary of forest plots, scatter plots, leave-one-out analyses, and funnel plots of causal associations between docosatrienoate (22:3n3) levels and rheumatoid arthritis.
